# Investigating the impact of motion visual synchrony on self face recognition using real time morphing

**DOI:** 10.1038/s41598-024-63233-2

**Published:** 2024-06-07

**Authors:** Shunichi Kasahara, Nanako Kumasaki, Kye Shimizu

**Affiliations:** 1 Sony Computer Science Laboratories, Inc., Tokyo, 141-0022 Japan; 2 Okinawa Institute of Science and Technology Graduate University, Okinawa, 904-0412 Japan

**Keywords:** Human behaviour, Perception

## Abstract

Face recognition is a crucial aspect of self-image and social interactions. Previous studies have focused on static images to explore the boundary of self-face recognition. Our research, however, investigates the dynamics of face recognition in contexts involving motor-visual synchrony. We first validated our morphing face metrics for self-face recognition. We then conducted an experiment using state-of-the-art video processing techniques for real-time face identity morphing during facial movement. We examined self-face recognition boundaries under three conditions: synchronous, asynchronous, and static facial movements. Our findings revealed that participants recognized a narrower self-face boundary with moving facial images compared to static ones, with no significant differences between synchronous and asynchronous movements. The direction of morphing consistently biased the recognized self-face boundary. These results suggest that while motor information of the face is vital for self-face recognition, it does not rely on movement synchronization, and the sense of agency over facial movements does not affect facial identity judgment. Our methodology offers a new approach to exploring the ‘self-face boundary in action’, allowing for an independent examination of motion and identity.

## Introduction

Self-face recognition serves as a cornerstone in constructing individual identity and fostering social relationships^1^. In the realm of visual communication, interventions on one’s face are prevalent, either through video communication or by using avatars^2,3^ as representations of oneself. Indeed, as a very idiosyncratic case, undergoing surgical procedures such as facial transplantation can lead to an update in one’s self-image^4^. How we identify ourselves and others affects trust and social communication^5–8^. With the emergence of computer-synthetic facial images, including high-quality AI-generated portraits, understanding how we recognize facial identities has become increasingly critical^9^. A series of research has indicated that self-face recognition is malleable and biased^10,11^. As reported in numerous studies, the judgments of self and others can transform through multisensory integration, including visuo-tactile,^12–19^, and visuo-motor^2,20,21^. Also known as an "enfacement illusion", participants start to identify with the other’s face, experiencing a shift in self-face recognition toward the other’s face through experiencing synchronous multisensory stimulation. Moreover, shifts in self-face recognition induced by multisensory stimulation have been reported to be influenced by various factors. For instance, the boundaries between self and others have been associated with social traits, perceptual and social binding, relationships, and attractiveness^13,14,18,22–25^, schizophrenia disorder^26,27^, personal bias^28^. These phenomena exhibit similarities with the sense of body ownership^29,30^, and are beginning to be elucidated in connection with neural activity^15,20,26,31^.

Previous studies have utilized morphing still images to investigate self-recognition, aiming to explore the boundaries between the self and others. Nevertheless, the majority of these studies have primarily used static images. This approach potentially overlooks a crucial dimension of self-recognition with action^32^—our association with moving images, which is analogous to observing our reflections in a mirror. Research indicates that facial identity recognition is not solely based on simple low-level visual features^33^. It also captures high-level features, including invariant and changeable aspects of faces^34^. However, the recognition of one’s face as a moving object has not been well explored. This limitation restricts our understanding to recognition through still images^12,13,35^.

Some experiments have employed visual stimuli using Computer Graphics (CG) and instructed participants to move, essentially generating motion-visual synchronization^2,20,21,36^. However, synthetic faces generated using CG are less natural than videos of faces, while natural videos of faces are ecological stimuli but provide limited control of facial form and motion. These tradeoffs highlight the difficulty in designing well-controlled dynamic face stimuli^32^.

Furthermore, while several studies have reported a correlation between the sense of agency and body ownership^37–41^, it remains unclear whether the sense of agency influences the visual identity of the body, especially face^42^. In other words, by investigating whether altering the state of motor-visual synchronization results in changes in the recognized facial identity, we can validate the relationship between motion agency and the visual identity of the face. Taken together, the influence of synchronization with one’s movements on self-face recognition remains an unexplored domain, necessitating further research to elucidate these dynamics.

In addressing this issue, we utilized the state-of-the-art image synthesis technology^43–46^ to create a new experimental paradigm. Our experiment system generated real-time morphing face video from the participants’ faces to those of others of the same gender, allowing changing facial identity. While controlling facial identity, the face image moves in sync with the participant’s facial movements to replicate the experience of viewing one’s reflection (Fig. 3).

This approach enables the exploration of the boundaries of self-face recognition not just through still images, but also in a state incorporating one’s facial movements. We investigated the impact of visual facial movements on the boundaries of self-face recognition through three conditions: Static, in which participants observed only morphing still images; Sync, in which the face images synchronized with one’s facial movements; and Async, in which there were movements, but they were not in sync with the facial images. Note that in the manuscript, we describe the boundary for self-other face recognition as the self-face boundary.

Regulating the similarity of the two faces has traditionally been challenging when generating morphing images between two faces^47^. This is due to the inherent difficulty in defining a common metric for facial distance, i.e., the distance between face identities^48–50^, as the morphing percentage becomes merely a relative metric, with the image information distance varying even among faces of the same race, whether they are similar or not. Previous research reported that there is a correlation between the distance in latent space and the perceptual and cognitive distance of two facial images^49,51^. Leveraging this property, we utilized the distance of latent vectors of the pre-trained GAN network^43^ as the metric of the distance between two faces for synthesizing a tailored ‘other’ face which will be at a constant distance in the latent space, from each participant’s face.

In this manuscript, we first validate our proposed face scale with six different morphing destination faces in experiment 1. Then, in experiment 2, we use the face morphing visual stimuli to investigate the categorical boundary between self and other. Participants stopped the visual stimuli with a keypress when they felt that the face looked more like themselves than the other and vice versa, depending on two conditions of the morphing direction. This study adopts a 2 × 3 within-subjects design to examine the interplay between motion-visual synchronization and self-face recognition. Then, we discuss our findings about the self-face boundary in action from the result of our experiment.

## Experiment 1

This experiment aims to validate our proposed facial morphing scale by examining how participants perceive self-recognition in response to morphing facial images using multiple target faces. Therefore, we designed the experiment to minimize biases from motion and morphing direction. By presenting static images in a randomized order, we ensure that the measurement of self-other boundaries is not influenced by the direction of morphing.

In the series of experiments conducted, we first describe the method used to generate the morphing facial stimuli. This includes a detailed explanation of how distances were defined within our proposed distance definition, ensuring that a series of morphing facial images maintain a constant distance. Subsequently, we investigate how participants perceive self-recognition in response to these morphing facial images. A schematic representation of the experimental design is depicted in Fig. 2a, with further details in the following paragraphs.

### Materials

**Figure 1:**
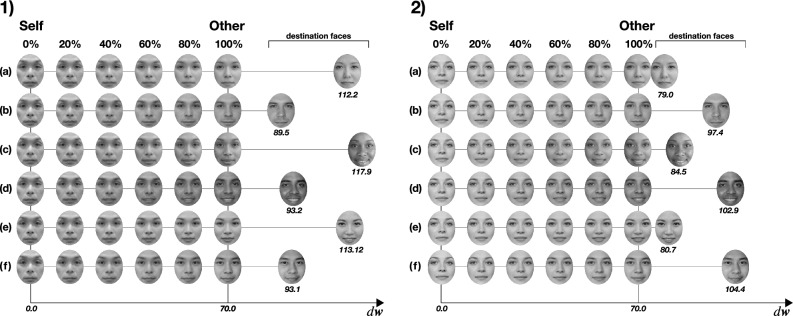
Visual stimuli were tailored individually for each participant by generating face morphing images from the participant’s face to the other face with 100 steps. Each 100% other face is tailored for each participant with a constant distance in the latent space from the participant’s face. (**a**–**f**) shows examples of morphing images with different morphing destinations. (1) is an example of an actual individual who is not a participant but of the same race as the participants. (2) is an example of a generated face image that does not exist.

Visual stimuli were tailored individually for each participant by generating a series of interpolating face morphing images that start with the participant’s face and end with the other face. Note that the self and the other face are tailored, but the destination faces are shared with participants. Figure 1 shows examples of tailored face morphing images. For synthesizing tailored other face images with a constant distance for each participant, we process images through the latent space of the pre-trained GAN network. Here, we denote that **w** is a vector in the intermediate latent space **W** which is a non-linear transformation of the **Z** space in StyleGAN pre-trained network^44^. In **W** space, the visual factors of variation become more linear and have disentangled representation. Investigation of Perceptual path length indicated that **W** is perceptually more linear than **Z** space. This allows us to linearly interpolate between the participant’s face and the predefined other faces.

The frontal image of their faces with a neutral expression was captured in the same environment with the same lighting conditions to acquire the participants’ faces. Adequate lighting was ensured, and participants were instructed to avoid wearing accessories or headgear. If the participant had hair obstructing the face, it was pinned back. The captured frontal face image was cropped to normalize face size with consistent posture. For other faces, we prepared six destination faces (a-f: three different races and two different genders) as the morphing destination 1. These images were generated from a StyleGAN2 pre-trained network^43,44^. These six destination face images were used consistently across participants.

To process images through the latent space, both the face of each participant (*i*) and the destination faces (*j* = *a, … , f*) were projected into StyleGAN2’s pre-trained latent space^45,52^, which estimates the closest latent vector **w**_self(*i*)_*,* **w**_destination(*j*)_ in the latent space. The projection method utilizes the LPIPS^53^ distance of the source and generated image in the loss function. Furthermore, the framework utilizes perceptual path length (PPL)^44^ to produce higher perceived overall image quality, leading to a higher quality face closer to that of the input facial image.

Given the same set of destination faces, the latent distance between **w**_self(*i*)_ and **w**_destination( *j*)_ is different for each participant *i*. Therefore, we linearly interpolate between the latent vector to generate **w**_other(*i, j*)_ that has a predefined distance from **w**_self(*i*)_. We calculated tailored latent vectors **w**_other(*i, j*)_ for generating the tailored other’s face from the latent vectors of the participant’s face **w**_self(*i*)_ and the destination face latent vectors **w**_destination( *j*)_.

$${\mathbf{w}}_{{{\text{other}}(i,j)}} = K \cdot \frac{{({\mathbf{w}}_{{{\text{destination}}(j)}} - {\mathbf{w}}_{{{\text{self}}(i)}} )}}{{\left| {{\mathbf{w}}_{{{\text{destination}}(j)}} - {\mathbf{w}}_{{{\text{self}}(i)}} } \right|}} + {\mathbf{w}}_{{{\text{self}}(i)}}$$

Here, *K* is a constant deviation from the participant, common across all facial samples from participants. The value of *K* = 70*.*0 is defined based on the preliminary investigation, as well as previous literature^51^. By using the obtained **w**_other(*i, j*)_ as the input into the generating network of StyleGAN2^43^, we can generate the tailored ‘other’ face which is a constant deviation from the participant toward each destination faces (Fig. 1).

One might consider the possibility of generating morphed images solely from projected vector data. However, in our preliminary experiments, when participants viewed the facial images generated using vectors obtained by projecting their own facial images into the StyleGAN2 network, they occasionally experienced a subtle sense of discomfort. This observation indicated that using the StyleGAN2 network alone might not reliably replicate the exact features of the participant’s face, potentially affecting the integrity of the 0% morphing (completely self) condition. Taking those aspects into account, we ensured that the morphing sequence always began with the participant’s actual facial image. We achieved this by blending the geometry and texture of the participant’s face with that of a generated ‘other’ face, derived from a point in the latent space at a certain distance. This approach allowed us to create morphing images that more accurately represented a continuum from ‘self’ to ‘other’. After generating the tailored other destination face, the facial image morphing process blended the mesh geometry and texture of the participant’s and the other faces. Facial feature point data were collected using MediaPipe framework and the segmented faces were treated as geometric points. These geometric point data and segmented textures were alpha-blended in relation with the morphing rate (0—100%). Lastly, a comprehensive spectrum of morphed faces was generated in 100 steps at 1 percent intervals for the experiment. Altogether, this approach enables the generation of morphing at a consistent distance in the latent space, despite variations in facial differences among participants.

### Experimental setup

Participants were comfortably seated in front of an LCD display (EX-LDGC251UT, 24.5 inch, 1920 × 1080 pixel, 60 Hz), and the distance of the eyes from the monitor was approximately 60 cm. In the experiment, the visual angle of the facial stimuli was 10.5 degrees. The screen height was adjusted in relation to the floor to resemble a mirror by aligning it with the participant’s head. The camera is mounted at the top of the display to capture the user’s face from a frontal view. The camera and the display are connected to a computer (CPU: AMD Ryzen 9 5950X 16-Core, GPU: NVIDIA RTX3090), which controls the experimental system and visual stimulus synthesis. The display was equipped with LED lighting to ensure the quality of the morphing face generation.

### Experimental procedure

Visual stimuli were tailored individually for each participant. Frontal pictures of participants’ faces with a neutral expression were taken with the camera, then 11 morphing images are generated (0% = self-face, 10, 20 …, 90, 100% = other-face) towards 6 different destination faces as shown in Fig. 1. Therefore, the total number of generated morphed face stimulus is 66 images. Note that 100% other faces with 6 different target faces are also tailored individually but with the consistent latent vector distance as defined as *K*. Participants were instructed to evaluate how much the faces displayed on the screen resembled themselves or someone else, utilizing a Visual Analogue Scale (VAS) with 0—100 scale. (How much does the image represent yourself on a scale of 0 = Self, 100 = Other). The trial was designed to involve responses to 66 morphed facial images, each being assessed five times, culminating in a total of 330 trials. It is important to note that the sequence of all trials was randomized. Participants were given a 30-s break after every 30 trials.

### Participants

A total of 30 participants with an average age of 27.0 (*SD* = 7*.*0) years participated in our study, of which 15 were female. All participants were of Asian ethnicity and resided in Japan, where the experiment was conducted. All participants in the two experiments reported here had either normal vision or vision that had been corrected using contact lenses; none wore glasses during the study. Participants signed an informed consent for study participation and were paid 20 dollar Amazon gift cards. The procedures were approved by the ethics committee of Sony Bioethics Committee and were following the ethical standards of the 1964 Declaration of Helsinki.

### Data collection

In the experiment, response data ranging from ‘self’ (0) to ‘other’ (100) were collected using the Visual Analogue Scale (VAS) for a total of 330 facial image trials. Additionally, before the experiment, participants answered their preferences for their own face with a Likert scale ranging from 1 to 7. We excluded data with the following criteria. The data from 1 participant who did not comprehend the method of responding to the experiment was excluded. Additionally, responses containing errors arising from artifacts in video synthesis were excluded. After these exclusions, the analysis was performed on the valid data comprising a total of 29 participants across 9570 trial response data.

## Results

**Figure 2:**
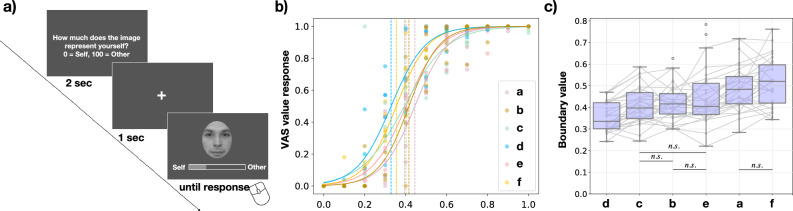
(**a**) The procedure of the trial for experiment 1. Participants were instructed to answer "How much does the image represent yourself?" on a scale of 0 = self, 100 = other. The VAS value was normalized as 0–1. (**b**) showcases a representative participant. The dashed lines represent the boundary value where VAS response reached 0.5. (**c**) Boundary values for each target face condition, arranged in ascending order of the average value. All pairs except labeled are significant.

For analysis, we conducted logistic regression analyses on the Visual Analogue Scale (VAS) responses (self = 0, other = 100, normalized as 0–1) for each participant’s morphing rate (0–100, normalized as 0–1) across six target face conditions. The morphing rate at which the VAS response reached 0.5 was defined as the boundary value. The overall mean McFadden’s pseudo-*R*^*2*^ for logistic regression was 0.54 (*SD* = 0*.*17). Figure 2b showcases a representative participant (P6, from the 20’s age, male). The logistic regression results for all participants are documented in the Supplementary Fig. S1. The boundary values across all target faces had a mean of 0*.*439 and a std of 0*.*111, with a minimum value of 0*.*221 and a maximum value of 0*.*783. Figure 2c displays the boundary values for each target face condition, arranged in ascending order of the average value for each target face.

To investigate the relationship between the target faces and the boundary values, A one-way repeated measures ANOVA was conducted to examine the effect of the target face condition on the boundary value. The analysis revealed a significant effect of the target face condition, *F*(5*,* 140) = 27*.*92*, p* < 0*.*001*.* Post-hoc comparisons using the Bonferroni correction indicated significant differences between pairs of target face conditions except for c–e (*p* = 0*.*768), b–e, a–f, and b–c (*p* = 1*.*000). These results suggest that there are significant differences in boundary values across different target faces, with only specific pairs of conditions showing non-significant contrasts.

In addition, a Mixed Linear Model (MixedLM) analysis was conducted to further evaluate the variability between subjects and the variability within subjects across different conditions, considering target face (d) as the intercept in the model. The intercept value of 0.356 indicates the estimated boundary value for ‘d’, with the coefficients (*β* ) representing differences in boundary values for each category. Significant effects were observed for all target face conditions compared to the reference target face ‘d’: e (*β* = 0*.*089*, p* < 0.0001), f (*β* = 0*.*160*, p* < 0.0001), c (*β* = 0*.*054*, p* < 0.001), a (*β* = 0*.*135*, p* < 0.0001), and b (*β* = 0*.*063*, p* < 0.0001). Notably, the random effects analysis indicated that the variance between subjects was estimated to be 0*.*006, suggesting a small degree of variability in the boundary value between different subjects.

To further investigate whether participant characteristics (age, gender) or individual preferences for their own face (*M* = 3*.*66*, SD* = 1*.*42*, min* = 1*, max* = 7) influence boundary values, we conducted two-way repeated measures ANOVA to examine the effects of gender, age, and self-face preference on boundary values across different target face conditions. The results indicated that the main effects of gender (*F*(1*,* 27) = 0*.*620*, p* = 0*.*438), age (*F*(14*,* 13) = 0*.*914*, p* = 0*.*567), and self-face preference (*F*(5*,* 23) = 1*.*481*, p* = 0*.*235) were not significant, and interactions with the target face conditions were also not significant. Overall, the target face condition significantly influences boundary values. However, the lack of significant interactions suggests that the effects of these individual characteristics on boundary values do not vary across different target face conditions.

### Discussion

The boundary values (*M* = 0*.*44*, SD* = 0*.*11, ranging from 0*.*22 to 0*.*78) indicated that the boundary value always fell within the range of morphing face images we designed. This suggests that setting *dw* = 70 as 100% of the other’s destination face allows for sufficient observation of the boundary between self and other within the facial distance (the latent vector distance *dw* = 70) used in this experiment. Particularly, between 20 and 60%, we observed a region where subjective self-face recognition and the morphing rate are approximately linear, as reported previously^47^, confirming the validity of this facial distance we proposed.

Furthermore, by using different target faces in this experiment, we evaluated the boundary value for morphing across different races and genders and assessed the variability between target faces and among participants. The results showed that while there is variability between target faces, the variability among participants is relatively small. Consistent with these findings, the additional analysis of the effects of participant gender, age, and individual preference for their own face on the boundary value revealed no significant main effects or interactions. This suggests robust trends in boundary values among participants. It is also necessary to recognize the fact that our participant group was sampled from the same ethnic group. However, our results indicate that when assuming the same ethnic participant group, it is valid to use a fixed target face to test the boundary value under different motion-visual conditions.

## Experiment 2

In this experiment, we investigate how motion-visual synchronization matters in self-face recognition. We designed the experiment to examine the boundary between self and others, with face-morphing visual stimuli based on the result of Experiment 1. The experiment was designed as a 2 × 3 within-subjects to investigate the interplay between motion-visual synchronization, and morphing direction on self-face recognition.

### Materials

The same as in experiment 1, visual stimuli were tailored individually for each participant. Frontal pictures of participants’ faces with neutral expressions were taken with the camera; then we generated 100 morphing images with a 1% step toward the destination faces corresponding to participants’ gender, as shown in Fig. 1. In this experiment, we used the face of Fig. 1b as the male destination face and Fig. 1a as the female destination face.

### Face morphing in action

**Figure 3:**
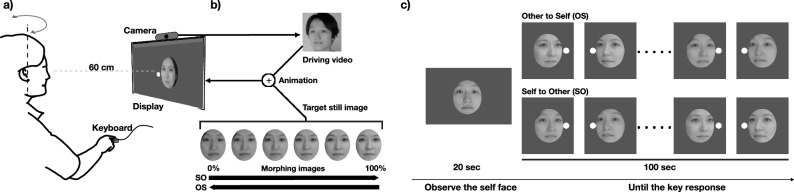
(**a**) Participants were instructed to rotate their faces periodically and immediately press a button when they felt the boundary of self-face in self to other (SO) condition or other to self condition (OS). (**b**) Real-time synthesis of videos that maintained the participant’s facial movements while morphing the visual identity of the face. (**c**) The procedure of the experiment trial. After 20 s of self-image exposure, participants started the face morphing task where participants were instructed to immediately press a key when they felt that the face looked "more like self than other" in the OS (other to self condition) and vice versa.

In the experiment, the system synthesizes a real-time morphing face video stimulus that moves in response to the participant’s facial movements, akin to a reflection in a mirror, with the previously generated morphing face images of each participant between the predefined other face (Fig. 3b). For real-time virtual mirror synthesis of moving face images, we employed the animation technology of Latent Image Animator^46^. This type of technology^46,54–56^ enables the animation of a still face image (source image) to move similarly to a moving face video (driving image) by using a self-supervised autoencoder and linear navigation in the latent space. We optimized the Latent Image Animator for real-time processing, using real-time camera input of the participants as the driving video, and progressively selecting the morphing face images series generated beforehand as the source image. This approach facilitated the synthesis of videos that maintained the participant’s facial movements while morphing the visual identity of the face.

In the Self to Other (SO) condition, we gradually set the morphing rate from 0 to 100%, thereby transitioning the images from Self to Other. Conversely, in the Other to Self (OS) condition, the morphing rate decreased gradually from 100 to 0%, resulting in a transition from other to self. This allowed the facial identity to morph while simultaneously reflecting the participant’s movements and expressions in real-time. The speed of morphing was set at 1% per second, with a 100% change occurring over 100 s. This speed was chosen by considering the speeds utilized in previous studies for examining self-boundaries^2,12,57^, further ensuring it was sufficiently slow so that any delays in response reactions would not significantly impact. Consequently, 100 s after the start of morphing, the face would completely transform into another person’s face if it started from Self, or into the participant’s own face if it started from Other.

Three further conditions were introduced for motion-visual synchronization: Sync, Async, and Static. In the Sync condition, participants observe the real-time face morphing in action as we described. Real-time processing was performed at approx. 30 FPS and the video delay from camera capture to display was approx. 250 ms, which is within the generally reported time delay for perceiving the sense of agency towards movement^58,59^. In the Async condition, to exclude the likelihood of perceiving some causality in motion due to uniform time delays, the delay of facial movement was smoothly and randomly varied between 320 and 2500 ms. In the Static condition, the participants’ movements were not reflected in the displayed face; participants only viewed a pre-generated morphing image as a still image.

In our experiment, including hairstyles in the facial images was technically possible. However, there was a potential confounding factor where judgments could be based on visual cues from the hairstyles. Therefore, to eliminate any synthesized artifacts from outside the facial region and to maintain consistency with previous studies^12,17^, we masked only the facial area from the chin to the forehead and converted the images to gray-scale images for visual stimuli.

### Experimental procedure

A schematic representation of the experimental design is given in Fig. 3c with a similar setup to experiment 1. Before conducting the main experiment, participants underwent a still photo shoot to create morphing images. In the first 20 s of each experiment trial, participants were instructed to look at the facial image with a self-image on the display. This procedure was for reconfirming one’s self-image (0% morphing) and to minimize the influence of previous trials, a practice that has also been carried out in past studies^60^. Subsequently, we started a trial to investigate the boundaries of self-face recognition during face morphing. In these trials, participants were instructed to periodically rotate their heads slightly by approximately 5.5° every 2 s, following a visual marker displayed on the screen (Fig. 3c). They were also instructed to look at the face displayed on the screen while rotating their head. Depending on the experimental condition, the face on the screen would start morphing either from self-face (0% morph) or from other-face (100% morph).

Participants were instructed to immediately press a button with the index finger of their dominant hand when they felt that the face looked "more like self than other" in the OS (other to self condition) or, "more like other than self" in the SO (self to other condition), depending on the morphing direction. Participants kept their index finger above the button to minimize the latency from judgment to reaction throughout the experiment. After each morphing sequence, participants responded to a questionnaire concerning their sense of agency (how much did you feel you control the face? 1–7) on the displayed facial image, followed by a one-minute break. This trial procedure was repeated two times for each of the six conditions, which were combinations of two morphing directions (Self-to-Other [SO], Other-to-Self [OS]) and three synchronization conditions (Sync, Async, and Static). The six conditions were organized into two blocks in a counterbalanced design, ensuring a variation in sequence both within the blocks and across participants, thereby avoiding any consecutive repetition of the same condition.

### Data collection

A total of 36 participants with an average age of 25.8 (*SD* = 6*.*32) years participated in our study, of which 15 were female. For participants, we followed the same procedure as experiment 1. In the experiment, depending on the direction of morphing, the timing when participants recognized the face as self or other was recorded as a morphing percentage (0–100%). The reported sense of agency towards the facial image in that trial was also collected. We excluded the data from 5 participants who did not comprehend the method of responding to the experiment. Additionally, responses containing errors arising from artifacts in video synthesis were excluded. After these exclusions, the analysis was performed on the valid data comprising a total of 31 participants across 368 trial data.

### Results

#### Self boundary

**Figure 4:**
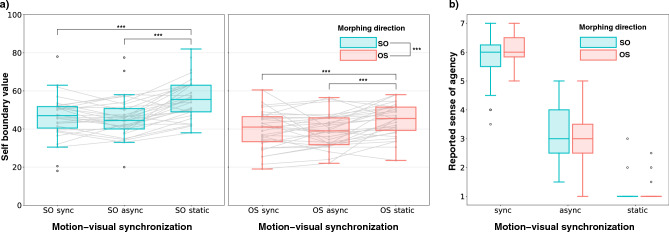
(**a**) Participant responses about self-face boundary in each condition, two directions: self to other (SO) and other to self (OS), and three motion-visual synchronization: sync, async, and static. (**b**) Participant response about the sense of agency score.

Figure 4a shows the participant response about self-face boundary in each condition. A two-way repeated measures ANOVA was conducted to examine the effects of *morphing direction* (direction: SO, OS) and *motion-visual synchronization* (Sync, Async, and Static) on participants’ percentage of the self boundary. There was a significant main effect of *morphing direction*, *F*(1*,* 30) = 10.626*, p* < 0*.*0001*, η*^2^ = 0*.*145. Additionally, there was a significant main effect of *motion-visual synchronization*, *F*(2*,* 60) = 27.645*, p* < 0*.*0001*, η*^2^ = 0*.*113. The interaction between *morphing direction* and *motion-visual synchronization* was insignificant, *F*(2*,* 60) = 2*.*6431*, p* = 0*.*079.

For post-hoc pairwise comparisons, we also performed Bonferroni-adjusted pairwise t-tests to compare participant responses between *morphing directions* and between *synchronization* conditions. The pairwise t-test for *morphing directions* showed a significant difference between OS and SO, *t*(92) =  *− *5*.*07*, p* < 0*.*001. For the *synchronization* conditions, there were significant differences between async and static, *t*(61) =  *− *6*.*74*, p* < 0*.*001, and between static and sync, *t*(61) = 6*.*19*, p* < 0*.*001. However, there was no significant difference between async and sync, *t*(61) =  *− *0*.*401*, p* = 0*.*69.

To ensure that our within-subject design across the six conditions did not potentially induce demand bias, we conducted a Mann–Whitney U test to compare the answer rates between the first and second halves of the experiment across all conditions. The results indicated that there were no statistically significant differences between the first and second halves in any of the conditions (all *p* > 0*.*20). This finding suggests that the participants’ performance remained consistent throughout the experiment, regardless of the condition.

#### Subjective scores

Here, we report the sense of agency on the displayed facial image as shown in Fig. 4b. A two-way repeated measures ANOVA was conducted to investigate the impact of *morphing direction* and *synchronization* on the perceived agency. The results indicated a significant main effect of *synchronization* on agency, *F*(2*,* 60) = 443*.*07*, p* < 0*.*0001*, η*^2^ = 0*.*885, but the main effect of *direction* was not significant, *F*(1*,* 30) = 0*.*021*, p* = 0*.*89. However, there was a significant interaction between *direction* and *synchronization*, *F*(2*,* 60) = 10*.*14*, p* < 0*.*001*, η*^2^ = 0*.*047. Further analysis within each *morphing direction* showed significant effects of *synchronization* for both OS, *F*(2*,* 60) = 478*, p* < 0*.*001*, η*^2^ = 0*.*912, and SO, *F*(2*,* 60) = 261*, p* < 0*.*001*, η*^2^ = 0*.*855. Pairwise comparisons using Bonferroni-adjusted t-tests revealed significant differences between all pairs of *synchronization* conditions within each direction (all *p* < 0*.*001). Additionally, analysis within each *synchronization* condition revealed a significant effect of *morphing direction* for async, *F*(1*,* 30) = 6*.*51*, p* = 0*.*016*, η*^2^ = 0*.*041, and sync, *F*(1*,* 30) = 9*.*54*, p* = 0*.*004*, η*^2^ = 0*.*065, but not for static. These results indicate that the agency scores are significantly influenced by the *synchronization* condition. The interaction between *synchronization* condition and *morphing direction* also suggests that the effect of synchronization on agency scores varies depending on the direction of interaction.

### Informed consent for facial images

In this manuscript, the facial images presented are not of the study participants. Instead, they are generated images of non- existent persons or actual images of the authors who have given their informed consent for the use of their images in this publication. All authors have been informed and have agreed to have their facial images published in an online open-access format, ensuring understanding and compliance with the publication’s use of their images.

## Discussion

From the two-way repeated measures ANOVA analysis on self boundary and the 2 × 3 factors, the findings suggest that both the morphing direction and motion-visual synchronization independently influence the self boundary. Furthermore, post-hoc analyses revealed differences in the self-face boundary due to variations in motion-visual synchronization. Specifically, the boundary of self-recognition was narrower when viewing moving faces (both in Sync and Async) than when viewing static images, implying that participants were more likely to broadly identify with static images of themselves than with their moving face image. These results may prompt a re-evaluation of previous studies on static image-based self-recognition. For instance, examining whether the shifts in self-recognition boundary observed in the enfacement illusion^12,15,20,21,31,61^ are also influenced by motion could offer insights into whether changes in self-recognition induced by multisensory integration are universal or specific to static images. This could further probe whether the transformations in self-recognition are generic to motion information. Additionally, these findings would provide significant social implications. Recognizing oneself based solely on static images might lead to a broader boundary of self-recognition, potentially embodying others within oneself and representing oneself more within others^14,17,62^. In contrast, recognizing oneself from moving images might result in a more stringent boundary of self-identification. The potential impact of these differences in self-face boundary due to the presence or absence of motion information on interpersonal relationships warrants further investigation. However, it is also possible that these differences in self-face boundary might stem from differential recognition efficacy between frontal and profile views, rather than the motion per se. Regarding this aspect, reports are suggesting that performance does not vary in recognizing others’ faces between these views^63^. Nonetheless, the design of our experiments could not disentangle whether the observed effects were primarily due to the information from the movement itself or the multiple facial postures brought about by head movement. Further research is needed to clarify these factors and their contributions to self-face recognition in dynamic contexts.

The absence of detected interaction in the analysis of the self-face boundary suggests that both OS and SO conditions consistently exhibit changes, irrespective of motion-visual synchronization. This implies that the presence or absence of motion information doesn’t merely introduce bias but rather independently contributes to judgments of self and others. Interestingly, in self-face judgments accompanied by motion, self-recognition boundaries were perceived as narrower for both SO and OS morphing. The mechanism behind this observation requires further experimental manipulation for elucidation. Indeed, the current experiment can be likened to a digital mirror test for self-recognition^29,64–66^. The neural mechanism of mirrored self-face recognition consists of several perceptual and semantic processes^64^. At the perceptual level, two independent processes detect different perceptual cues from a mirrored self-image. The contingency cue relates to the temporal alignment between one’s intentional facial action and the visually observed movement feedback, encompassing mirror recognition^66^ and the sense of agency associated with perceived action^67,68^. The figurative cue, on the other hand, is the congruence between the perceived face and the self-face representation stored in visual long-term memory. However, the development of this figurative cue process is believed to have evolved from the agency-related experience derived from the contingency cue^69^. At the cue integration stage, the belief-validation process evaluates the consistency between different self-relevant inputs at a perceptual level, such as between contingency and figurative cues. The cue integration process might explain the narrower self-recognition boundaries observed under conditions accompanied by motion. The existence of an optimal cue integration process for understanding others’ facial identities, considering both facial form and motion, has been suggested^70^. Hence, adapting the current methodology would bring the potential to extend these studies to self-identity recognition.

The elucidation of facial recognition mechanisms, especially the interplay between facial motion and identity recognition, has been a focal point of research for a long time^32,71,72^. Yet, the role of motion in both expression and identity recognition remains incompletely disentangled^73^. Previous reports suggest that observing a familiar face in motion aids in identity recognition^74^, indicating that motion contributes to the understanding of another’s identity. Moreover, it has been reported that recognizing identity solely from motion is possible^75^. Experimental evidence also shows a clear advantage for recognizing unfamiliar faces when they move non-rigidly, as compared to static faces, highlighting the undeniable link between facial motion and identity recognition^76^. On the other hand, the uniqueness of self-face recognition has also been debated. For instance, from facial image inversion experiments, it has been posited that the advantage in self-face processing might be attributed more to the engagement of self-related attentional mechanisms than mere familiarity^77^. This notion is further supported by event-related potential (ERP) studies^78^, suggesting that the self-face is recognized differently than just a highly familiar face. In light of this debate on the distinctiveness of self-face recognition, our methodology, which allows for the modulation of identity while retaining other elements, holds the potential to contribute to those questions.

From our study, the main effect observed for motion and visual synchronization on agency underscores the pivotal role that the synchronization of motion and visual cues plays in influencing the sense of agency, aligning with expectations^37,79^. Taking this result into account, and considering the observation that both Sync and Async motion-visual synchronizations did not significantly impact the self-face boundary responses, several insights can be drawn. While past research has indicated that the sense of body ownership (the feeling that "this is my body") is enhanced by motion-visual synchronization^37,38,40^, our findings hint at the possibility that facial identity and the sense of facial ownership might operate through distinct processes. This inference resonates with prior report^29^. Furthermore, the significant interaction observed between "direction" and "motion and visual synchronization" in the sense of agency accentuates the collective influence of these variables on agency perception. This suggests that the underlying identity’s starting point could potentially influence the experienced sense of agency. In other words, while the sense of agency might not directly influence identity recognition, the inverse might hold true: the recognition of identity could impact the sense of agency. With these controlled experimental setups, there’s an anticipated potential for more advanced analyses concerning the interplay between the sense of agency and identity.

## Conclusions

Our experiment uniquely achieved the examination of self-face identity recognition accompanied by motion under varying visual-motor synchronization conditions, while independently modulating facial identity. This investigation revealed that self-face recognition with motion exhibits different characteristics from static self-face. Furthermore, the absence of substantial differences between Sync and Async conditions clarified that self-motion synchronization doesn’t significantly influence self-face identity recognition. The methodologies introduced in this study, particularly the means to explore the ‘self-face boundary in action’, provide a novel approach and an opportunity for a fresh revisit to the realm of self-face recognition research, allowing for the independent examination of motion and identity.

### Supplementary Information

Supplementary Figure 1.

## Data Availability

The datasets generated and analyzed during the current study are available in the OSF repository (https://osf.io/c2p95/).
