# Supplementary material for: Investigating the impact of motion visual synchrony on self face recognition using real time morphing
Source: Sci Rep. 2024 Jun 7;14:13090. doi: 10.1038/s41598-024-63233-2 (PMC11161490; doi:10.1038/s41598-024-63233-2)
Supplement: Supplementary file 1 — Supplementary Figure 1. [file 41598_2024_63233_MOESM1_ESM.pdf]

Supplementary Figure S1

The logistic regression results for all participants in the experiment 1.

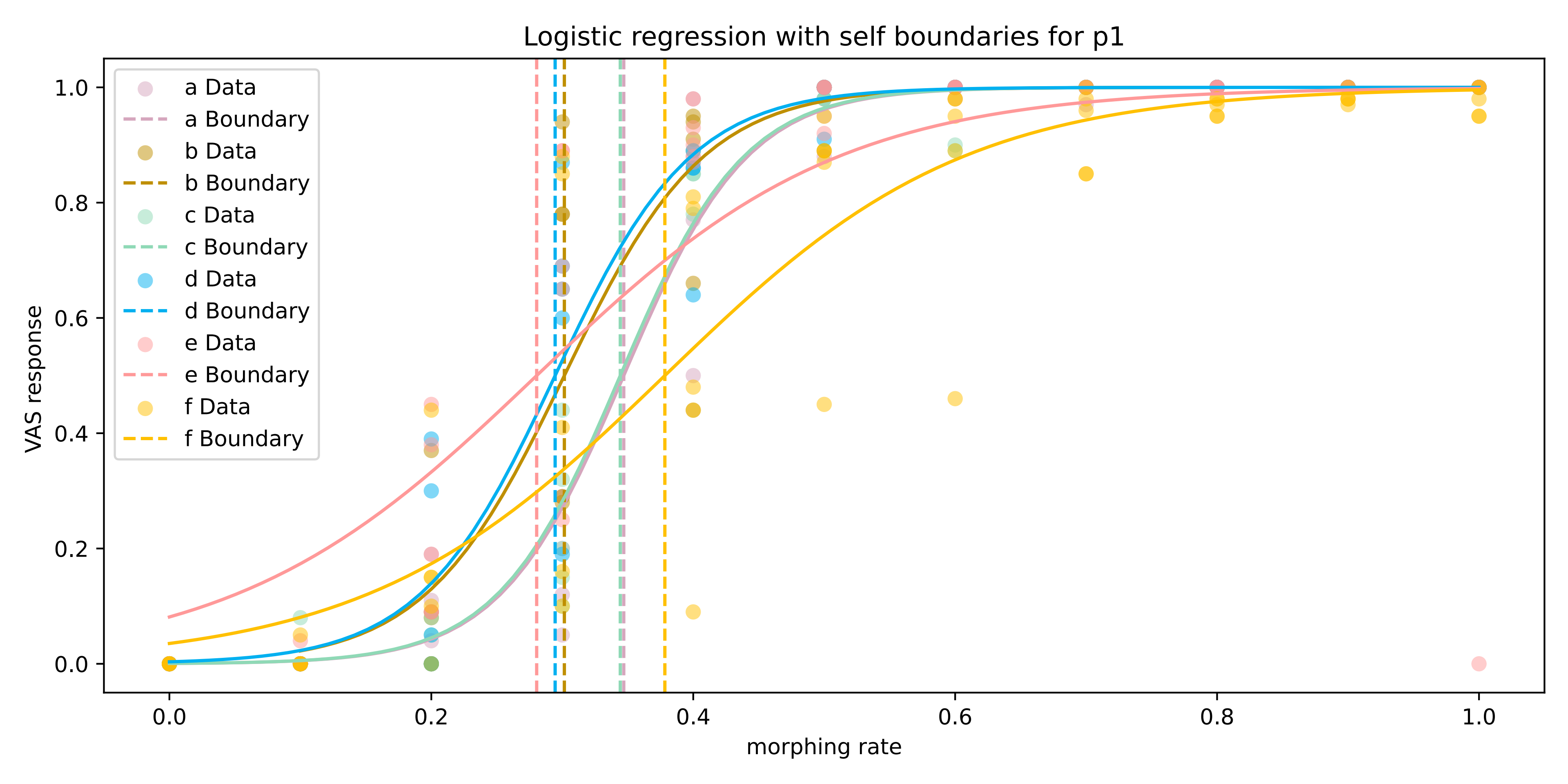

Logistic regression with self boundaries for p1
a Data
a Boundary
b Data
b Boundary
c Data
c Boundary
d Data
d Boundary
e Data
e Boundary
f Data
f Boundary
VAS response
morphing rate
0.0
0.2
0.4
0.6
0.8
1.0

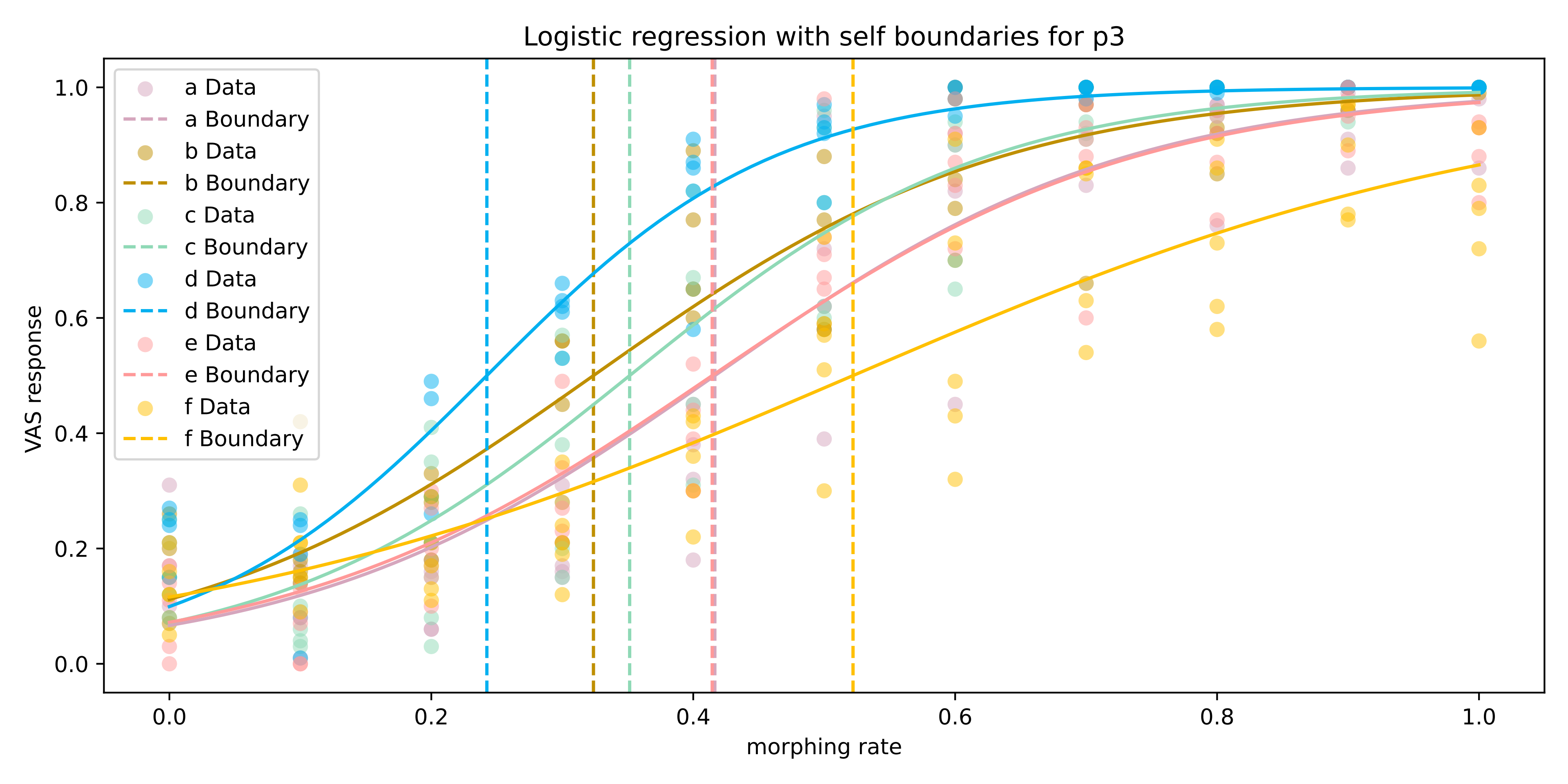

Logistic regression with self boundaries for p3
a Data
a Boundary
b Data
b Boundary
c Data
c Boundary
d Data
d Boundary
e Data
e Boundary
f Data
f Boundary
VAS response
morphing rate
0.0
0.2
0.4
0.6
0.8
1.0

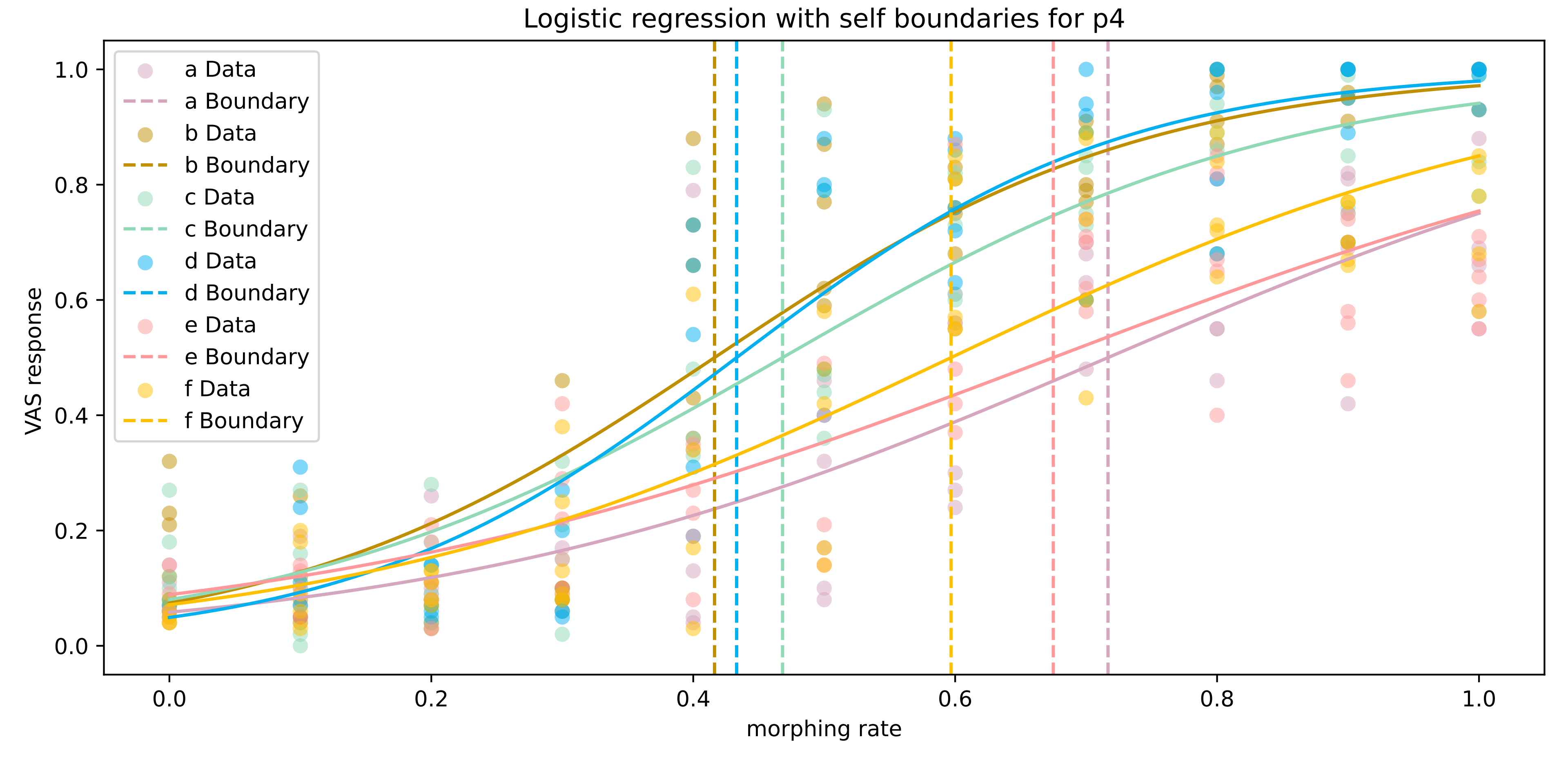

Logistic regression with self boundaries for p4
a Data
a Boundary
b Data
b Boundary
c Data
c Boundary
d Data
d Boundary
e Data
e Boundary
f Data
f Boundary
VAS response
0.0
0.2
0.4
0.6
0.8
1.0
morphing rate
0.0
0.2
0.4
0.6
0.8
1.0

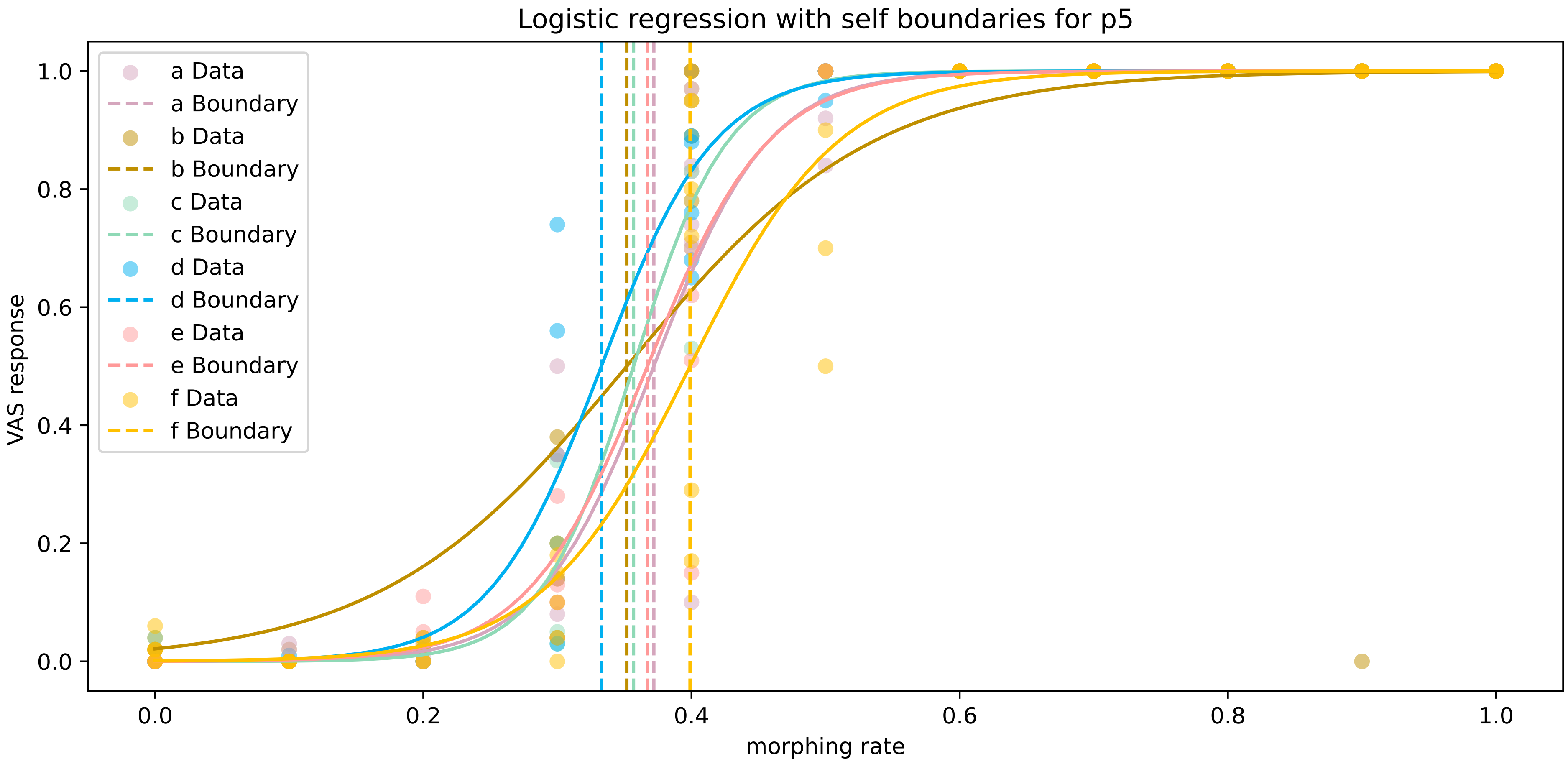
Logistic regression with self boundaries for p5
a Data
a Boundary
b Data
b Boundary
c Data
c Boundary
d Data
d Boundary
e Data
e Boundary
f Data
f Boundary
VAS response
0.0
0.2
0.4
0.6
0.8
1.0
morphing rate
0.0
0.2
0.4
0.6
0.8
1.0

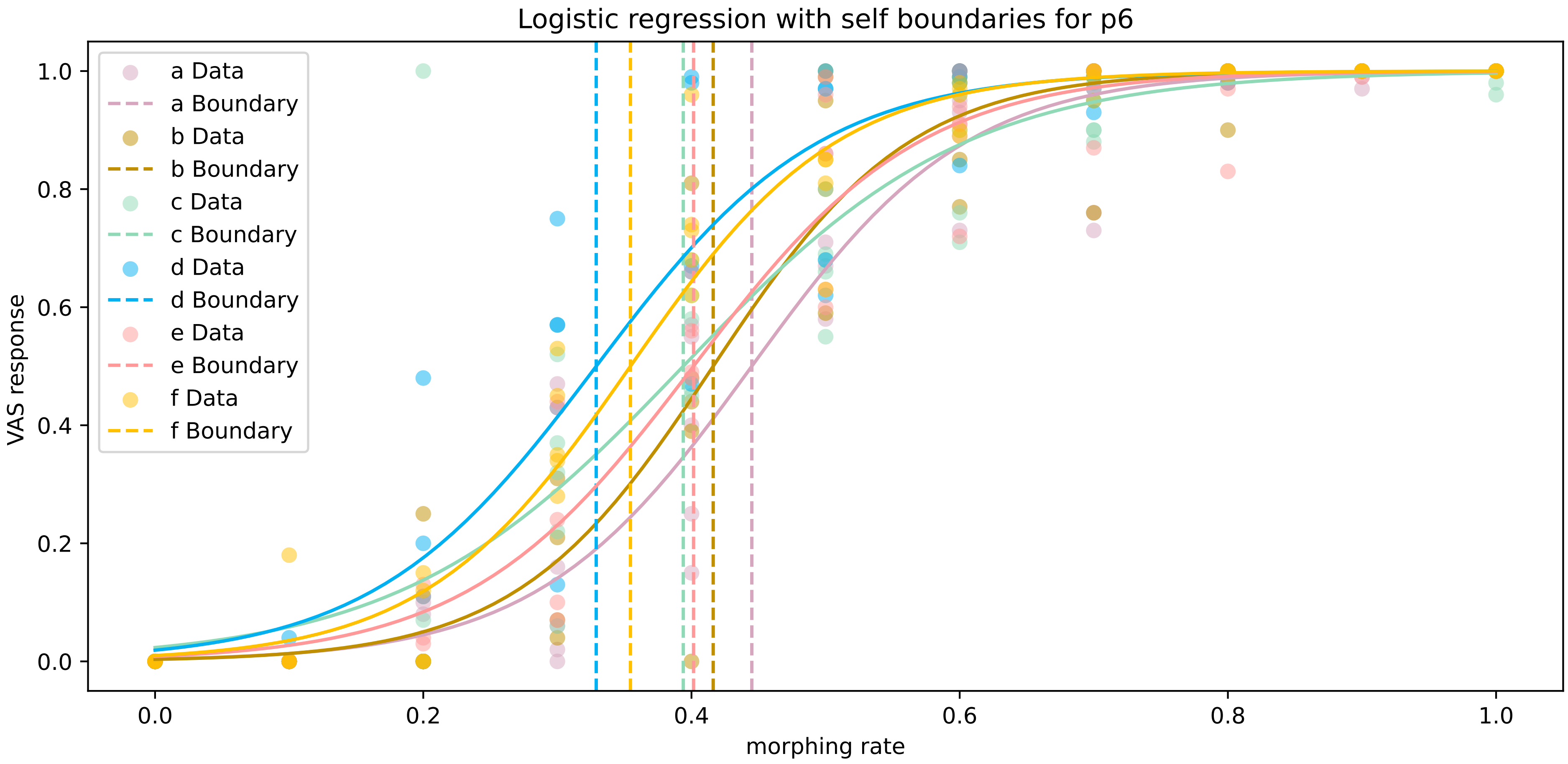
Logistic regression with self boundaries for p6
a Data
a Boundary
b Data
b Boundary
c Data
c Boundary
d Data
d Boundary
e Data
e Boundary
f Data
f Boundary
VAS response
1.0
0.8
0.6
0.4
0.2
0.0
0.0
0.2
0.4
0.6
0.8
1.0
morphing rate

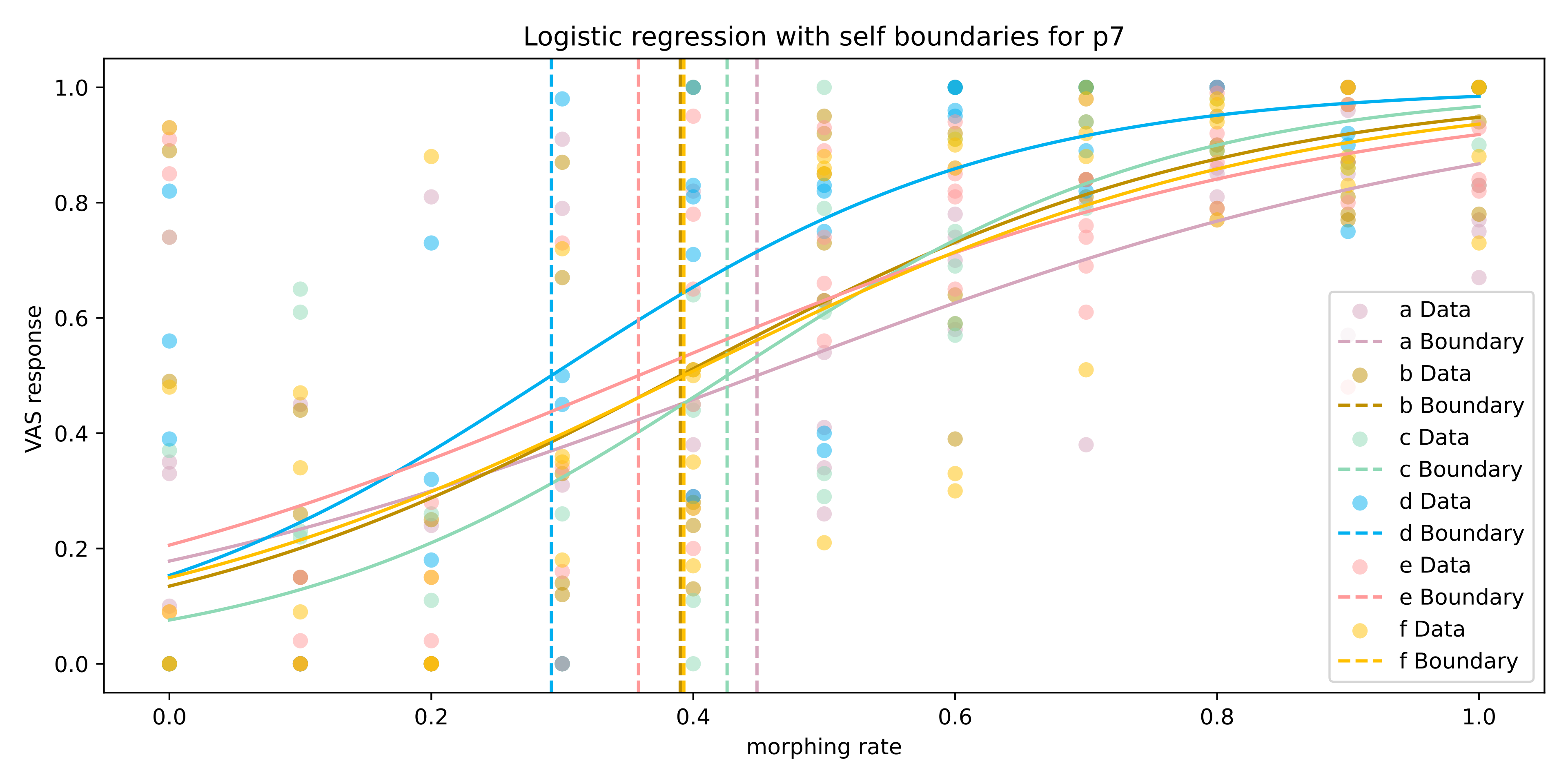

Logistic regression with self boundaries for p7
VAS response
morphing rate
0.0
0.2
0.4
0.6
0.8
1.0
a Data
a Boundary
b Data
b Boundary
c Data
c Boundary
d Data
d Boundary
e Data
e Boundary
f Data
f Boundary

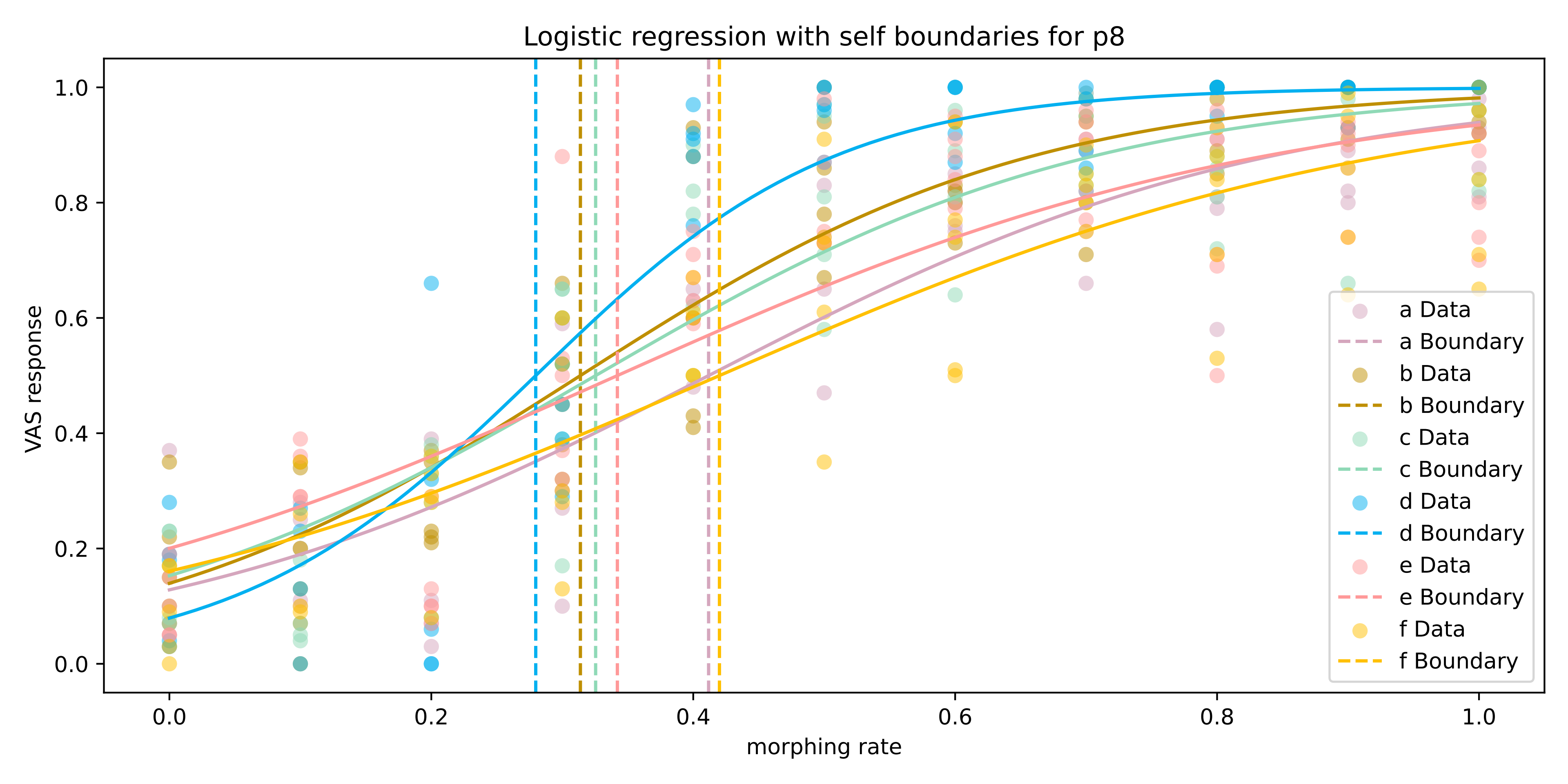
Logistic regression with self boundaries for p8
VAS response
morphing rate
0.0
0.2
0.4
0.6
0.8
1.0
a Data
a Boundary
b Data
b Boundary
c Data
c Boundary
d Data
d Boundary
e Data
e Boundary
f Data
f Boundary

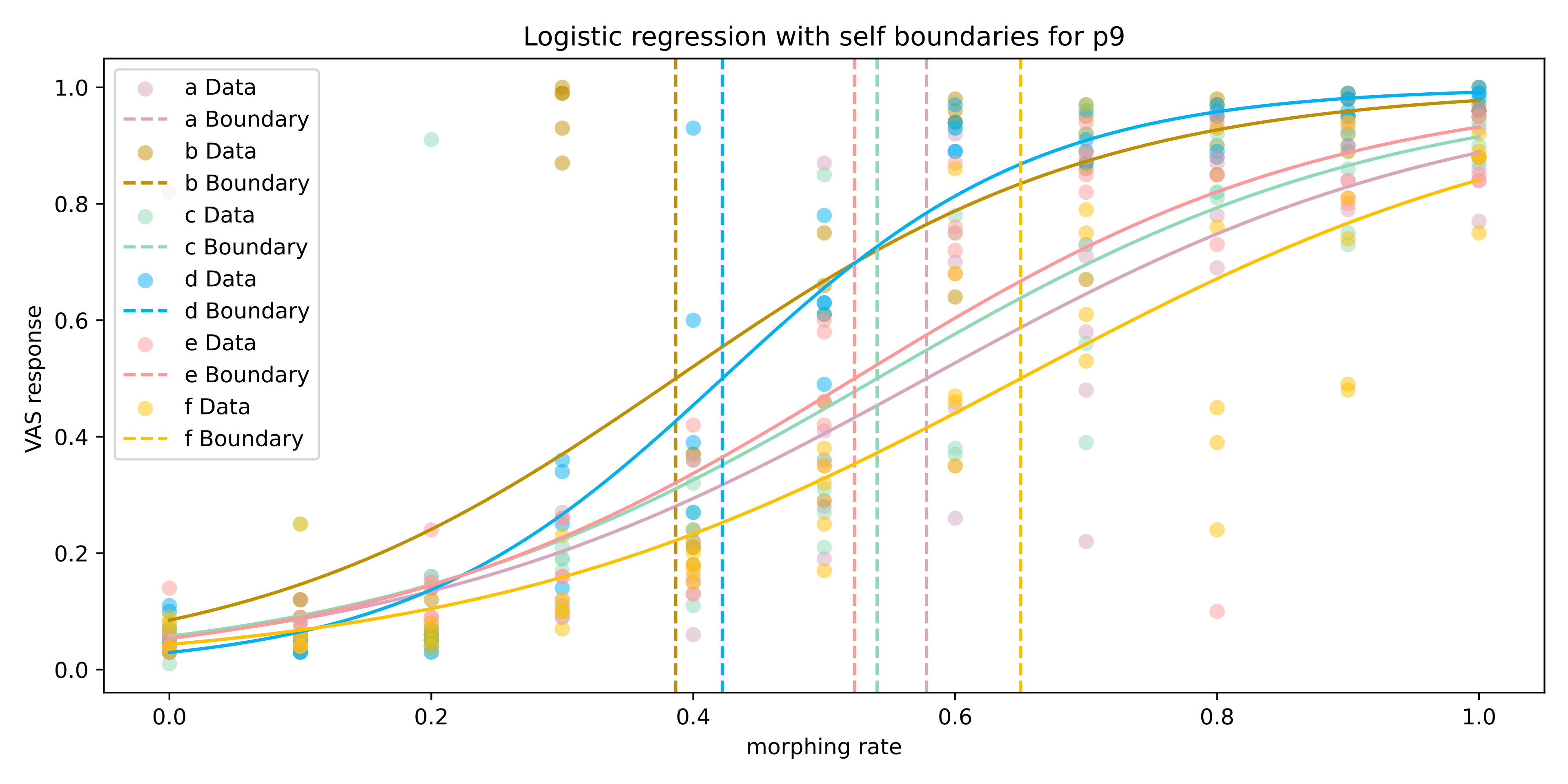

Logistic regression with self boundaries for p9
a Data
a Boundary
b Data
b Boundary
c Data
c Boundary
d Data
d Boundary
e Data
e Boundary
f Data
f Boundary
VAS response
morphing rate
0.0
0.2
0.4
0.6
0.8
1.0

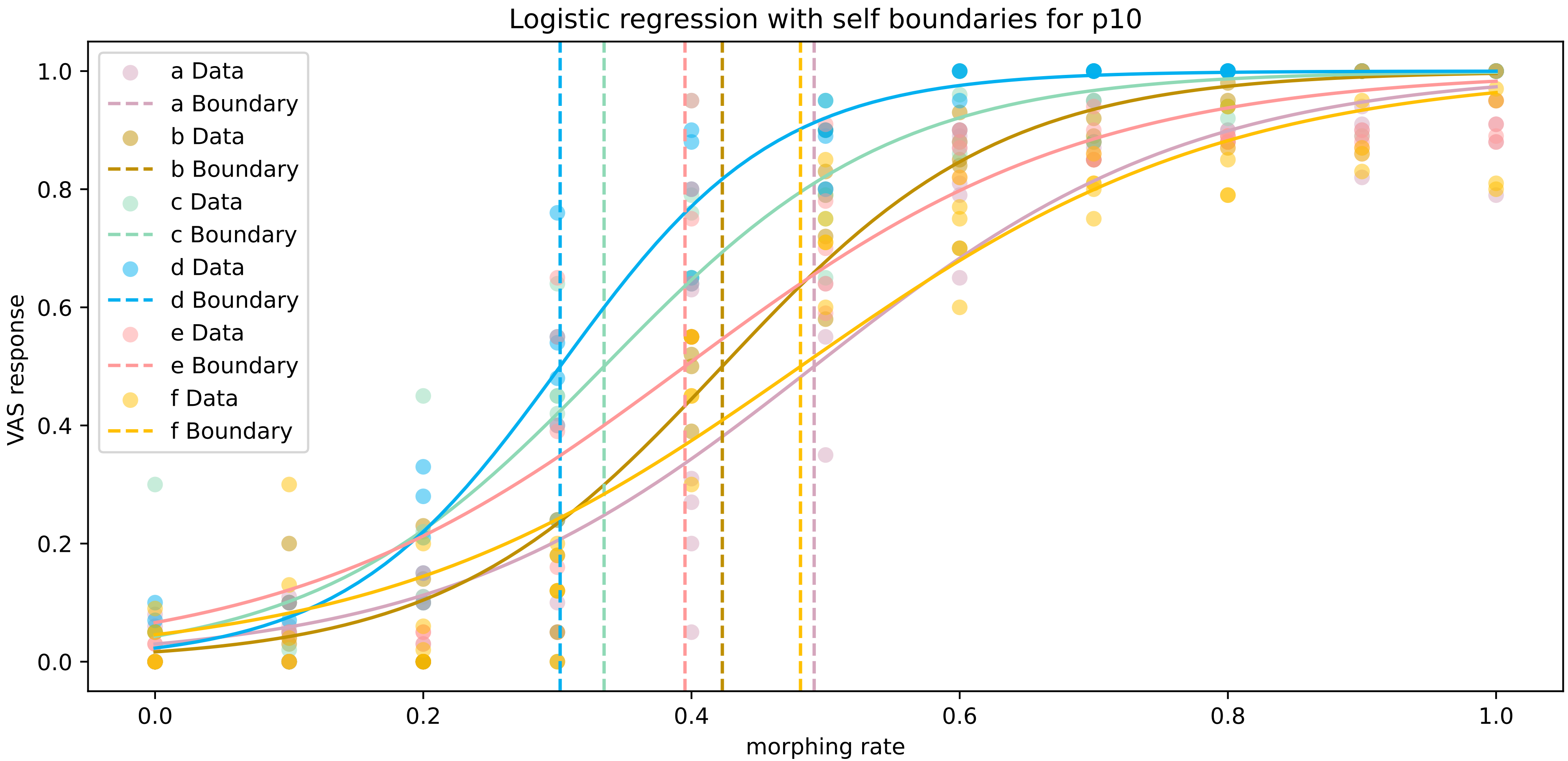

Logistic regression with self boundaries for p10
a Data
a Boundary
b Data
b Boundary
c Data
c Boundary
d Data
d Boundary
e Data
e Boundary
f Data
f Boundary
VAS response
0.0
0.2
0.4
0.6
0.8
1.0
morphing rate
0.0
0.2
0.4
0.6
0.8
1.0

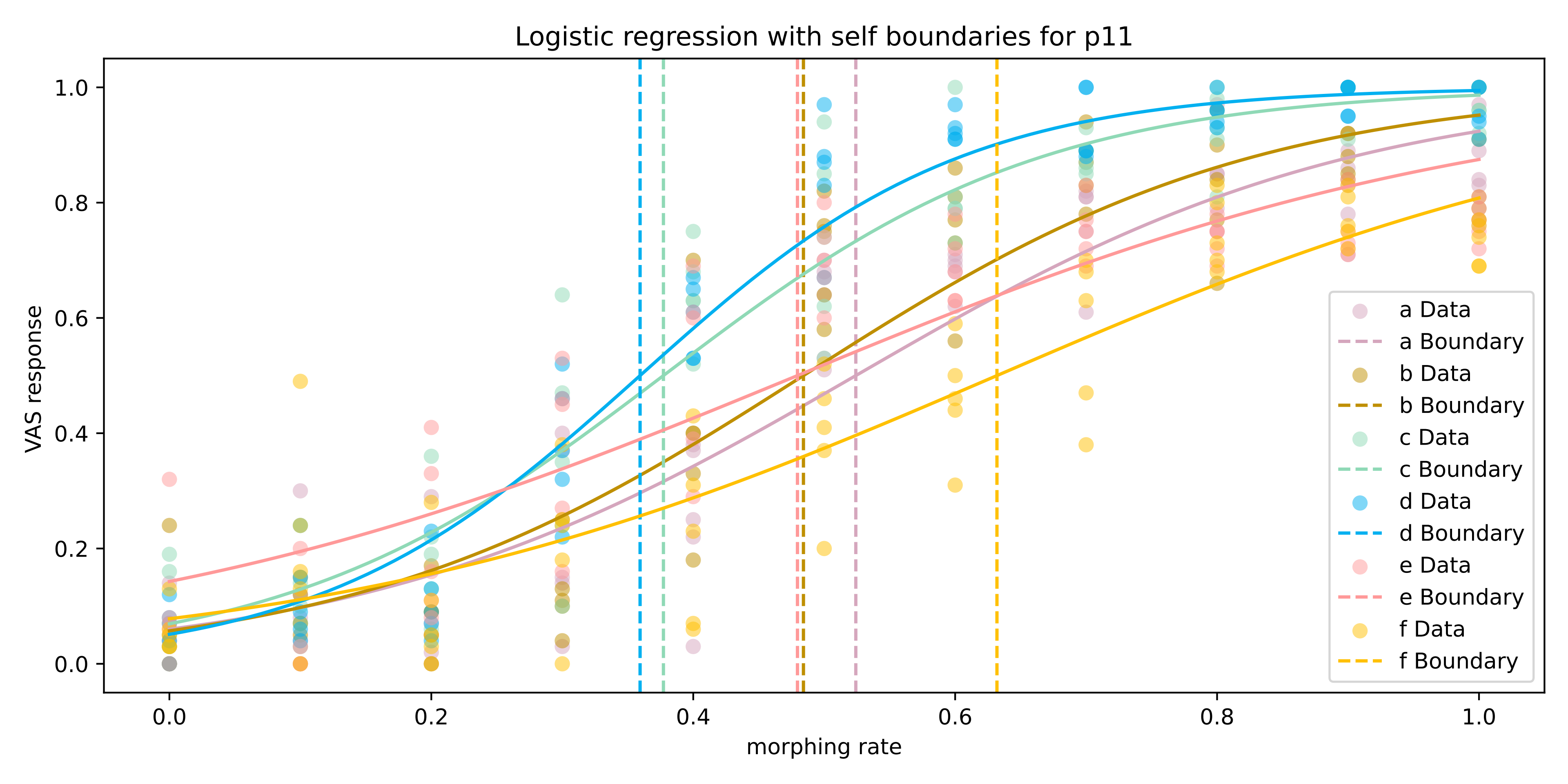
Logistic regression with self boundaries for p11
VAS response
morphing rate
0.0
0.2
0.4
0.6
0.8
1.0
a Data
a Boundary
b Data
b Boundary
c Data
c Boundary
d Data
d Boundary
e Data
e Boundary
f Data
f Boundary

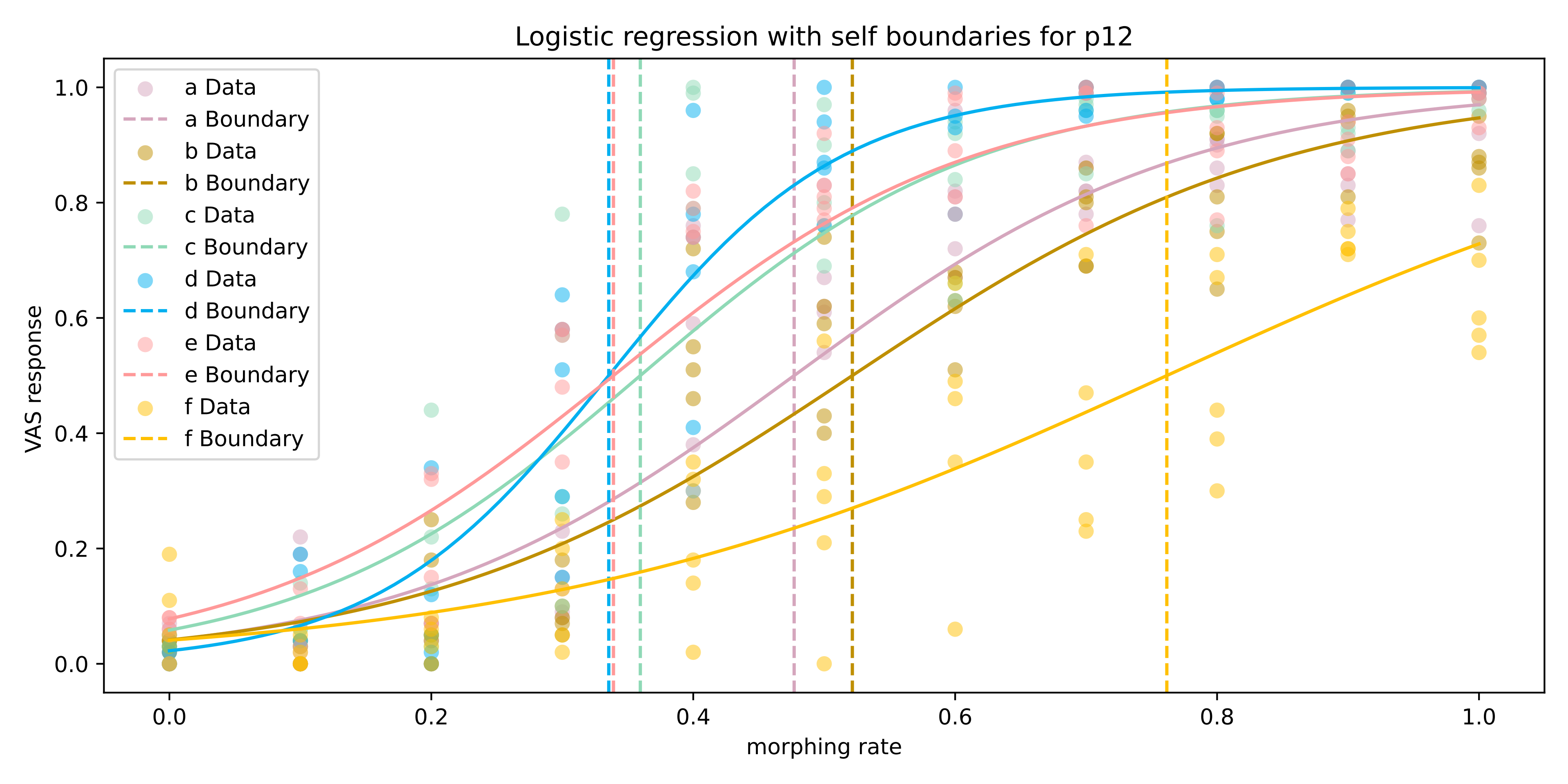

Logistic regression with self boundaries for p12
a Data
a Boundary
b Data
b Boundary
c Data
c Boundary
d Data
d Boundary
e Data
e Boundary
f Data
f Boundary
VAS response
morphing rate
0.0
0.2
0.4
0.6
0.8
1.0

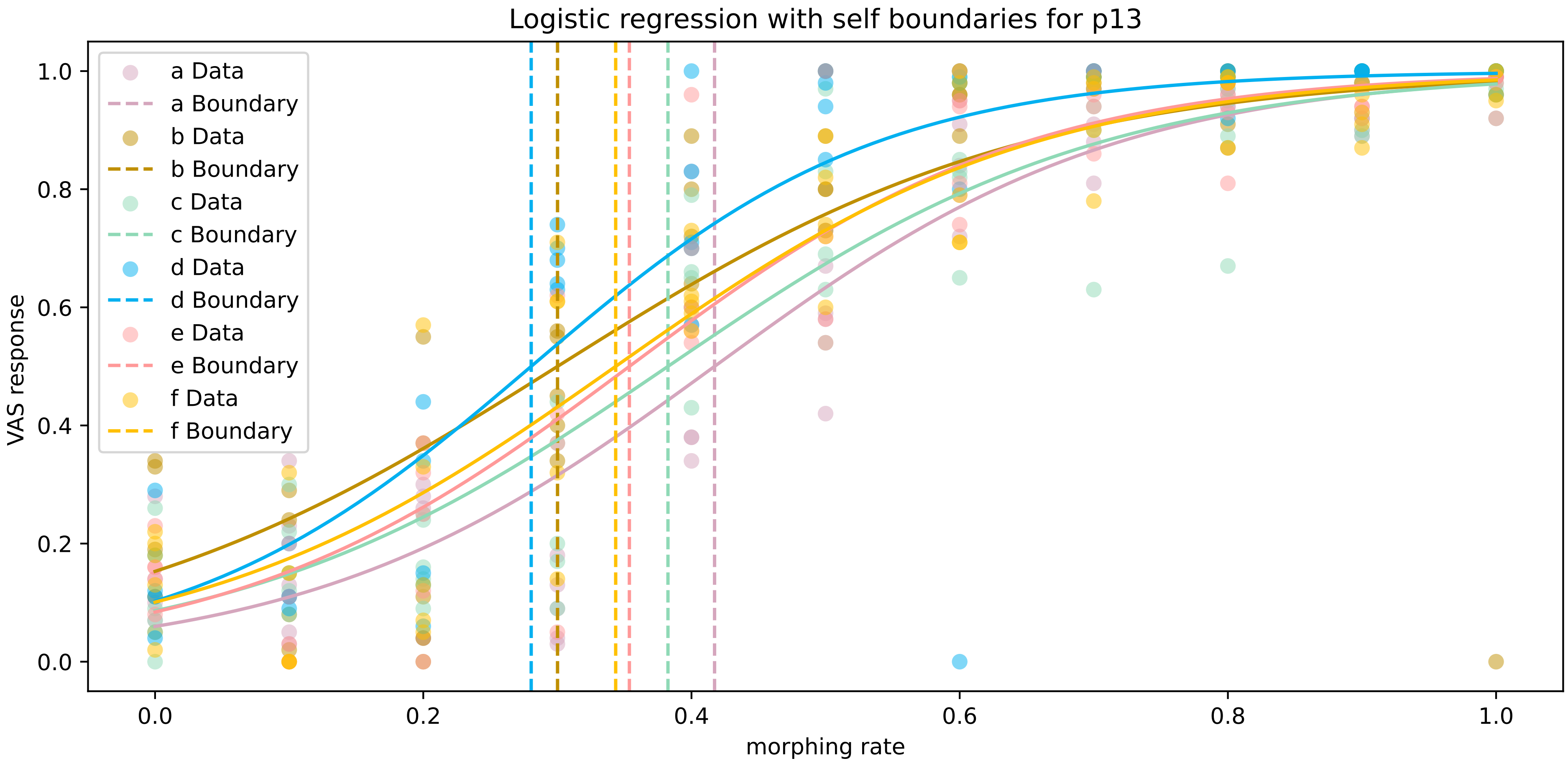

Logistic regression with self boundaries for p13
a Data
a Boundary
b Data
b Boundary
c Data
c Boundary
d Data
d Boundary
e Data
e Boundary
f Data
f Boundary
VAS response
morphing rate
0.0
0.2
0.4
0.6
0.8
1.0

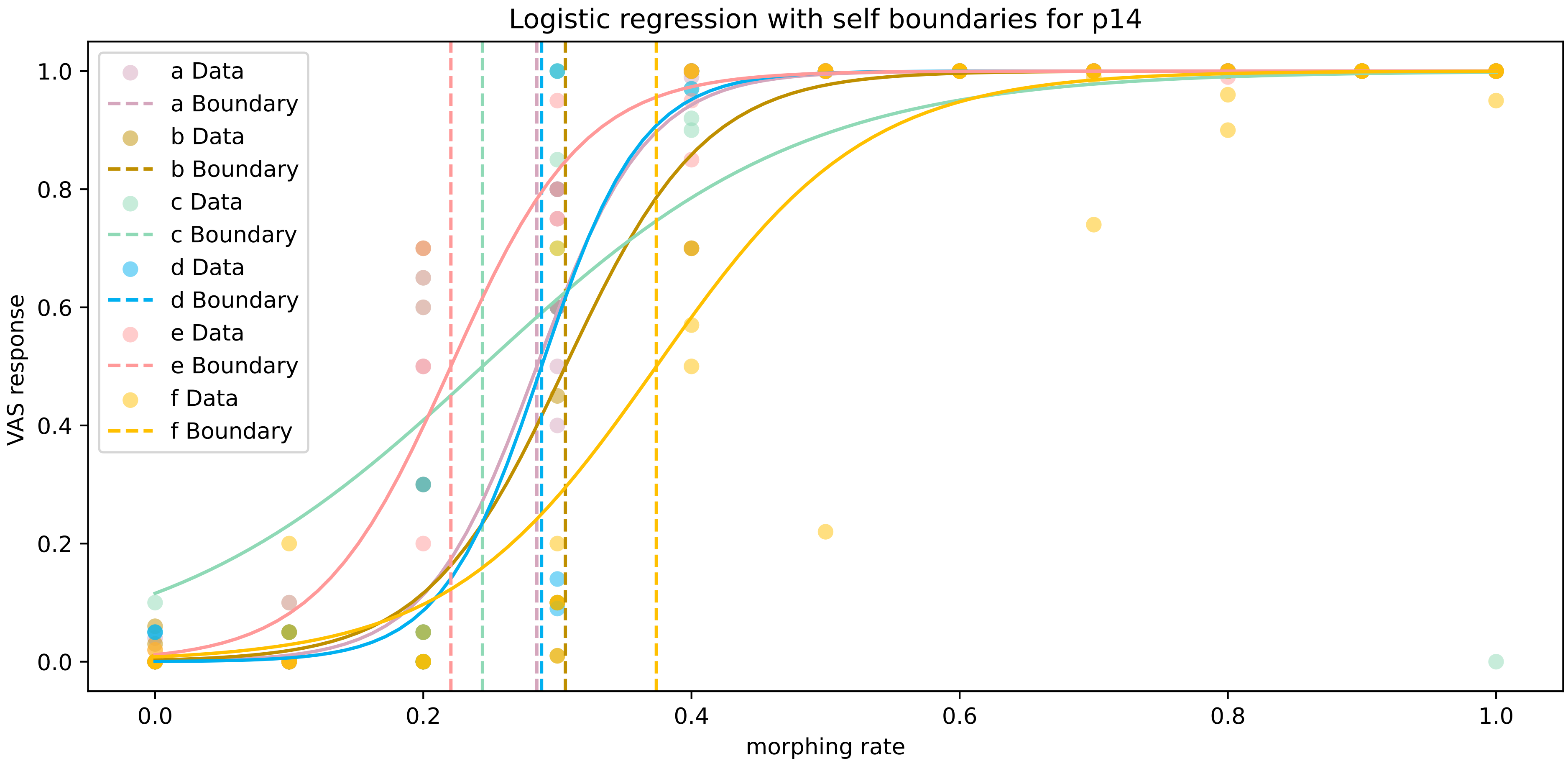

Logistic regression with self boundaries for p14
a Data
a Boundary
b Data
b Boundary
c Data
c Boundary
d Data
d Boundary
e Data
e Boundary
f Data
f Boundary
VAS response
morphing rate
0.0
0.2
0.4
0.6
0.8
1.0

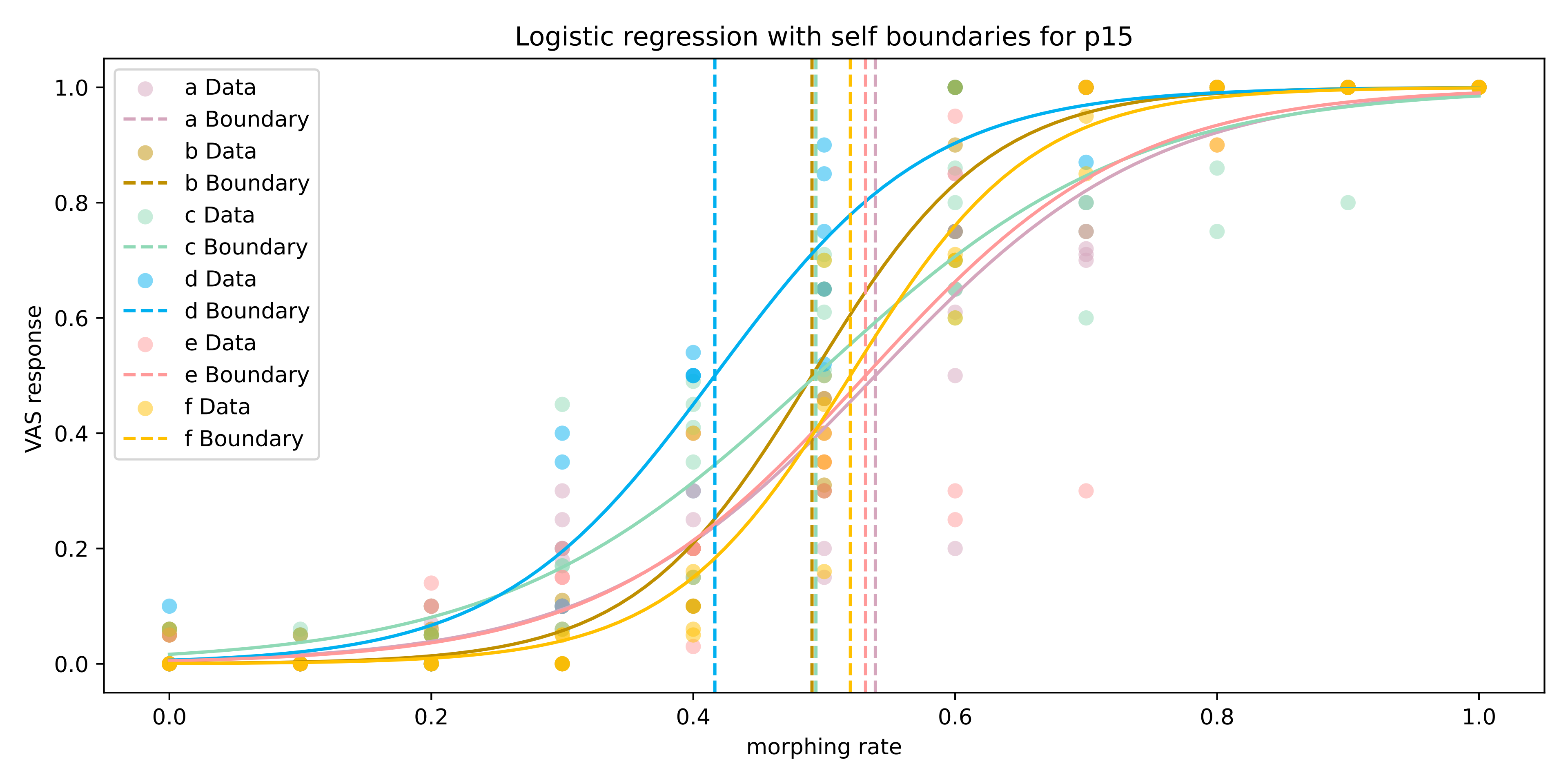

Logistic regression with self boundaries for p15
a Data
a Boundary
b Data
b Boundary
c Data
c Boundary
d Data
d Boundary
e Data
e Boundary
f Data
f Boundary
VAS response
morphing rate
0.0
0.2
0.4
0.6
0.8
1.0

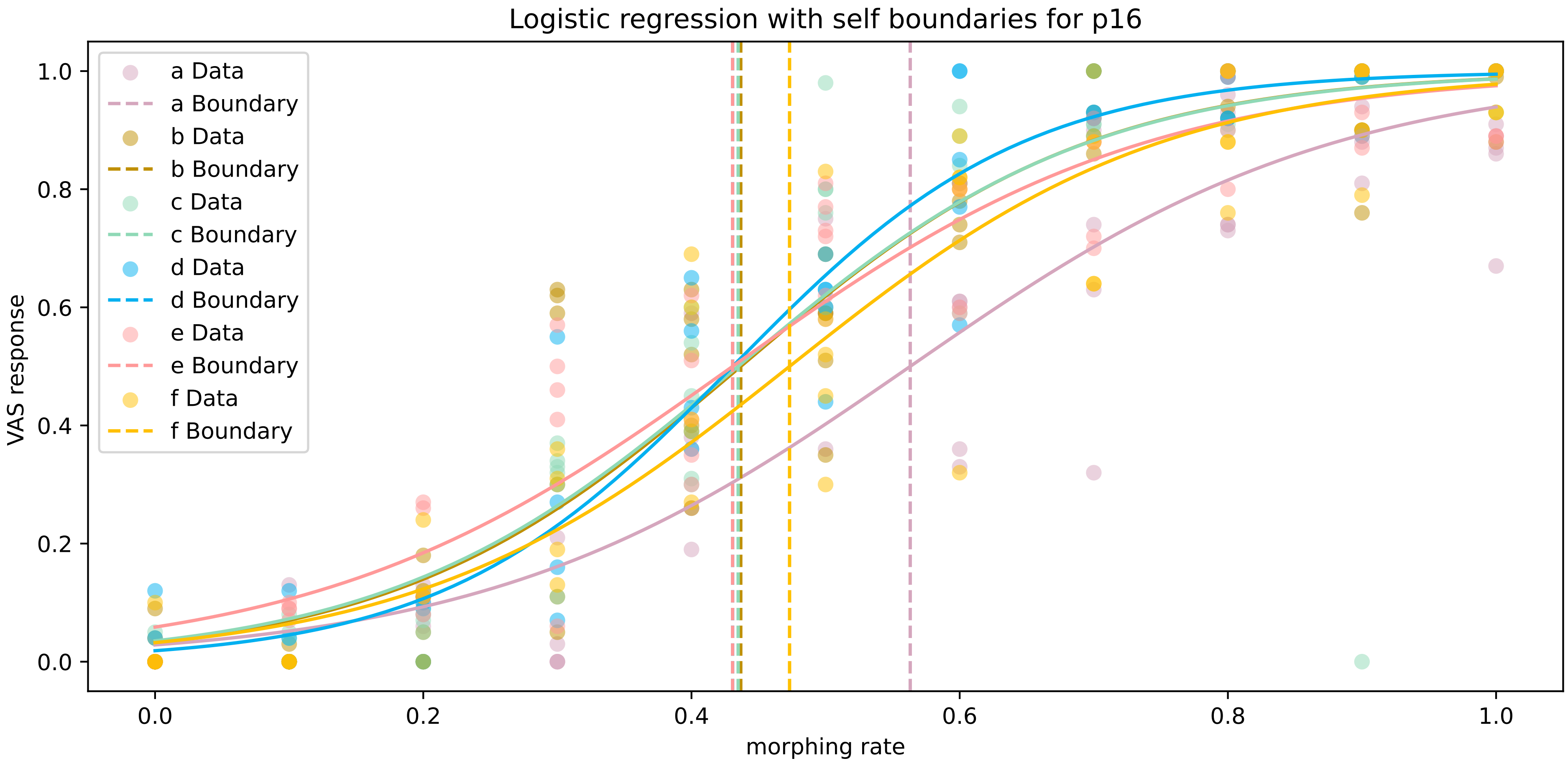
Logistic regression with self boundaries for p16
a Data
a Boundary
b Data
b Boundary
c Data
c Boundary
d Data
d Boundary
e Data
e Boundary
f Data
f Boundary
VAS response
morphing rate
0.0
0.2
0.4
0.6
0.8
1.0

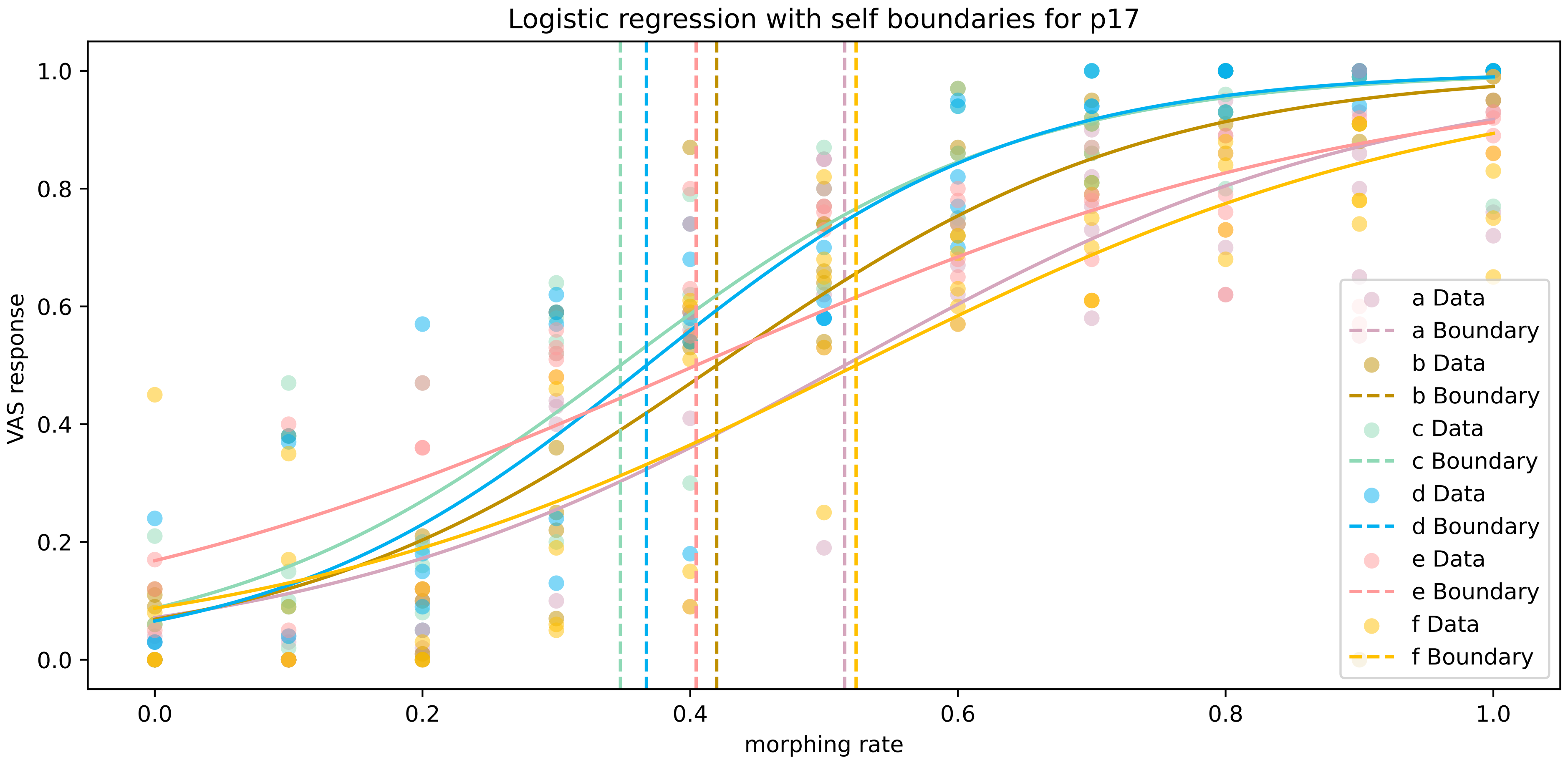

Logistic regression with self boundaries for p17
VAS response
morphing rate
0.0
0.2
0.4
0.6
0.8
1.0
a Data
a Boundary
b Data
b Boundary
c Data
c Boundary
d Data
d Boundary
e Data
e Boundary
f Data
f Boundary

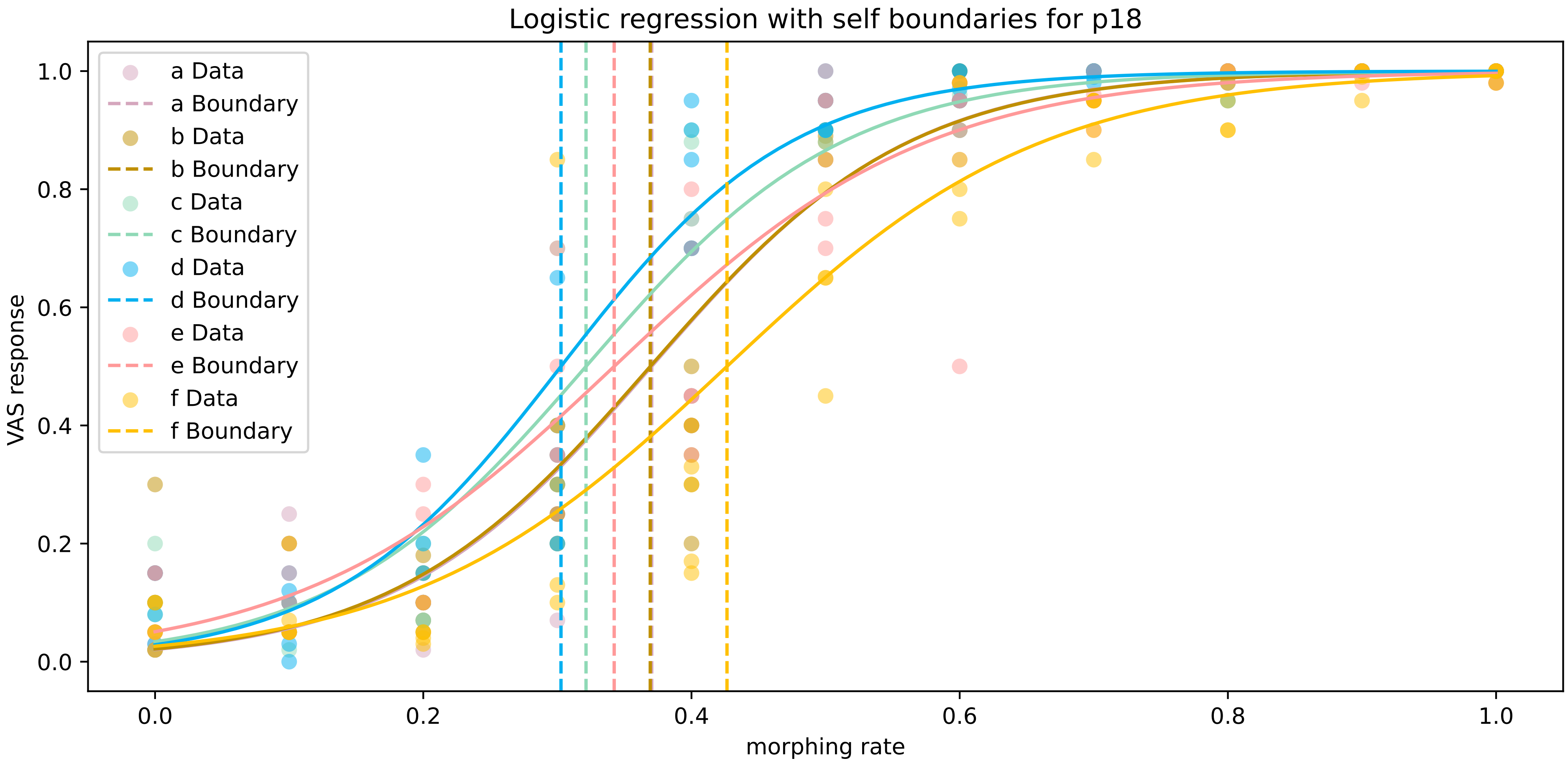

Logistic regression with self boundaries for p18
a Data
a Boundary
b Data
b Boundary
c Data
c Boundary
d Data
d Boundary
e Data
e Boundary
f Data
f Boundary
VAS response
morphing rate
0.0
0.2
0.4
0.6
0.8
1.0

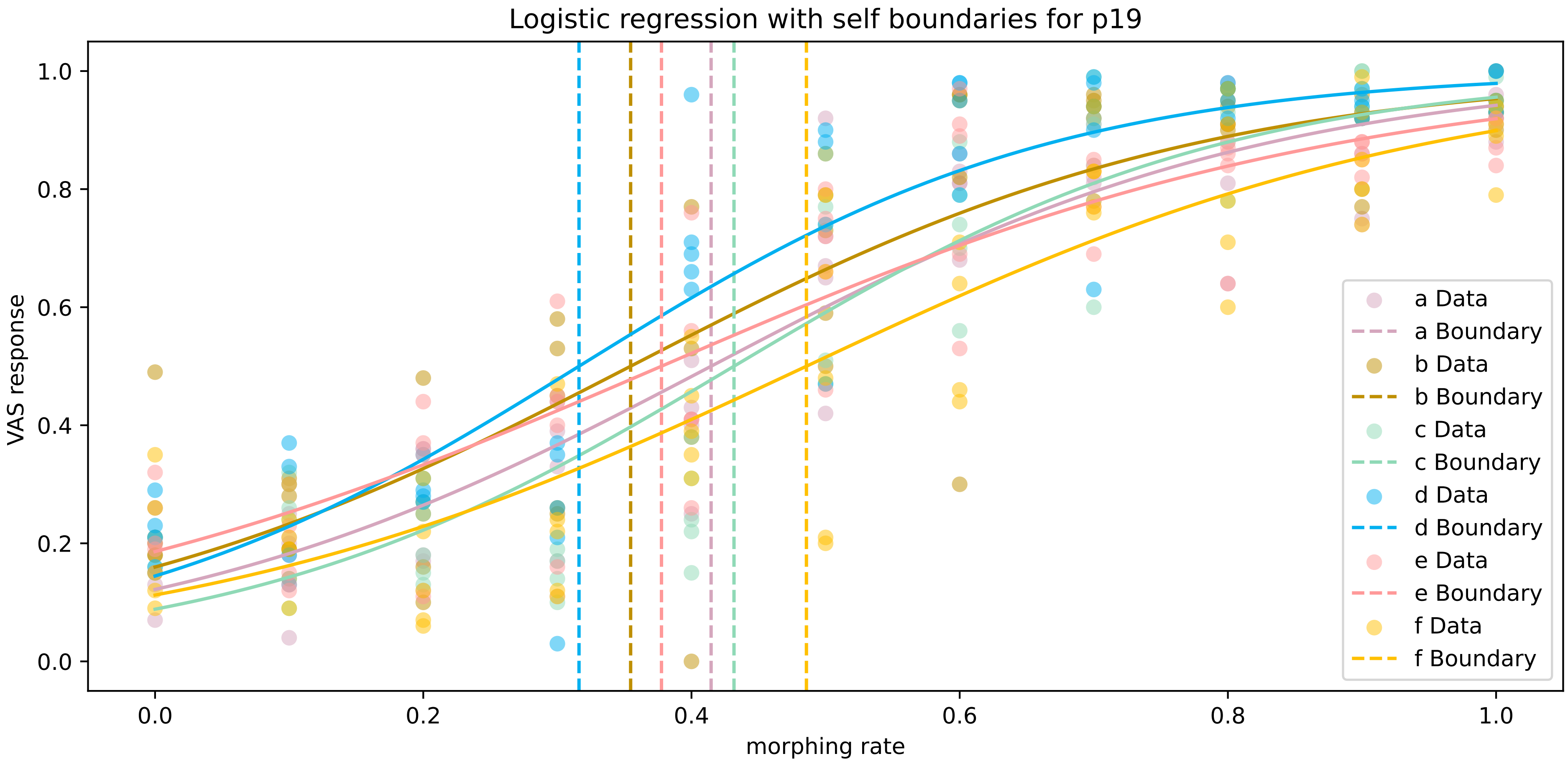

Logistic regression with self boundaries for p19
VAS response
morphing rate
0.0
0.2
0.4
0.6
0.8
1.0
0.0
0.2
0.4
0.6
0.8
1.0
a Data
a Boundary
b Data
b Boundary
c Data
c Boundary
d Data
d Boundary
e Data
e Boundary
f Data
f Boundary

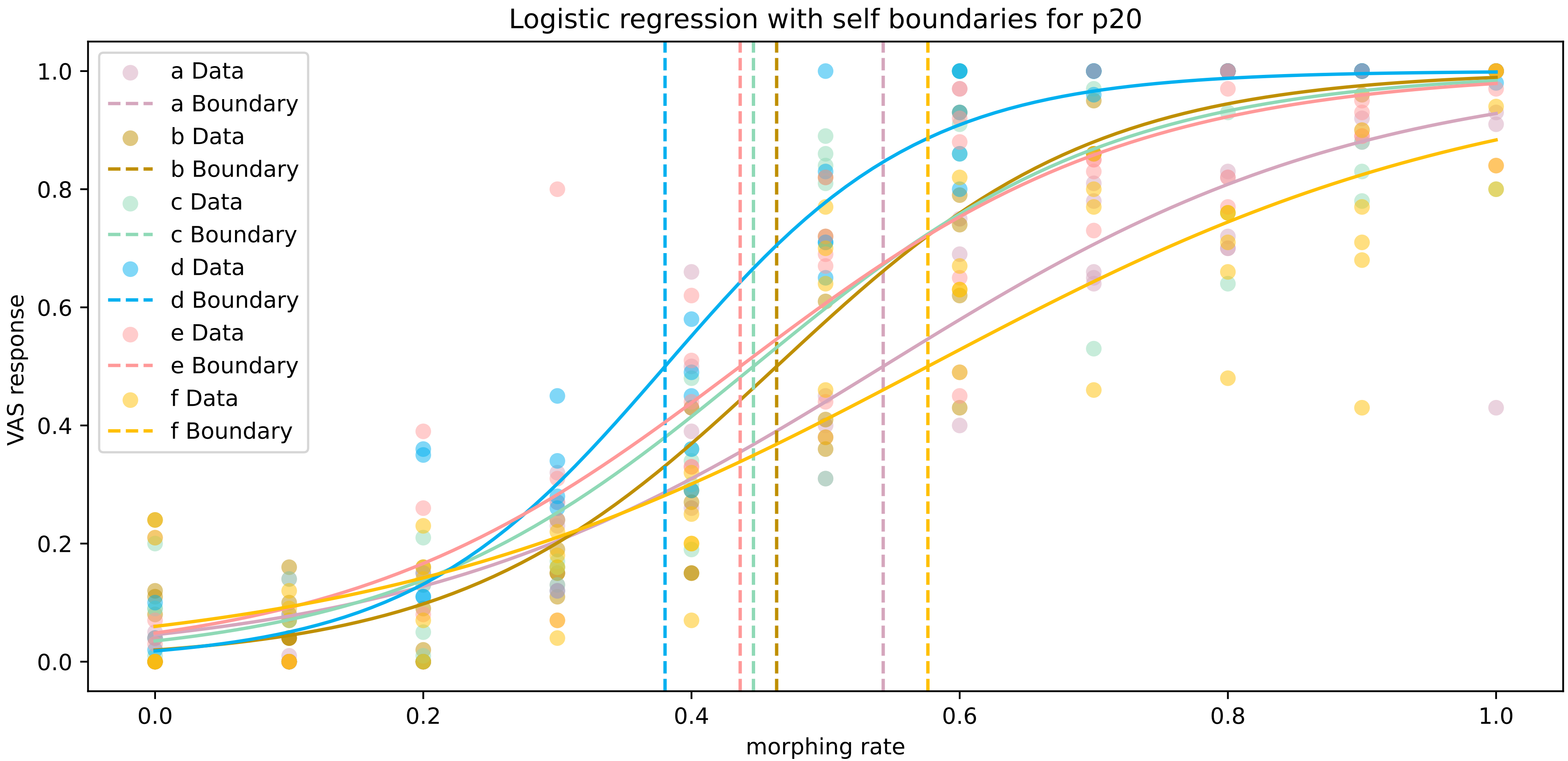

Logistic regression with self boundaries for p20
a Data
a Boundary
b Data
b Boundary
c Data
c Boundary
d Data
d Boundary
e Data
e Boundary
f Data
f Boundary
VAS response
morphing rate
0.0
0.2
0.4
0.6
0.8
1.0

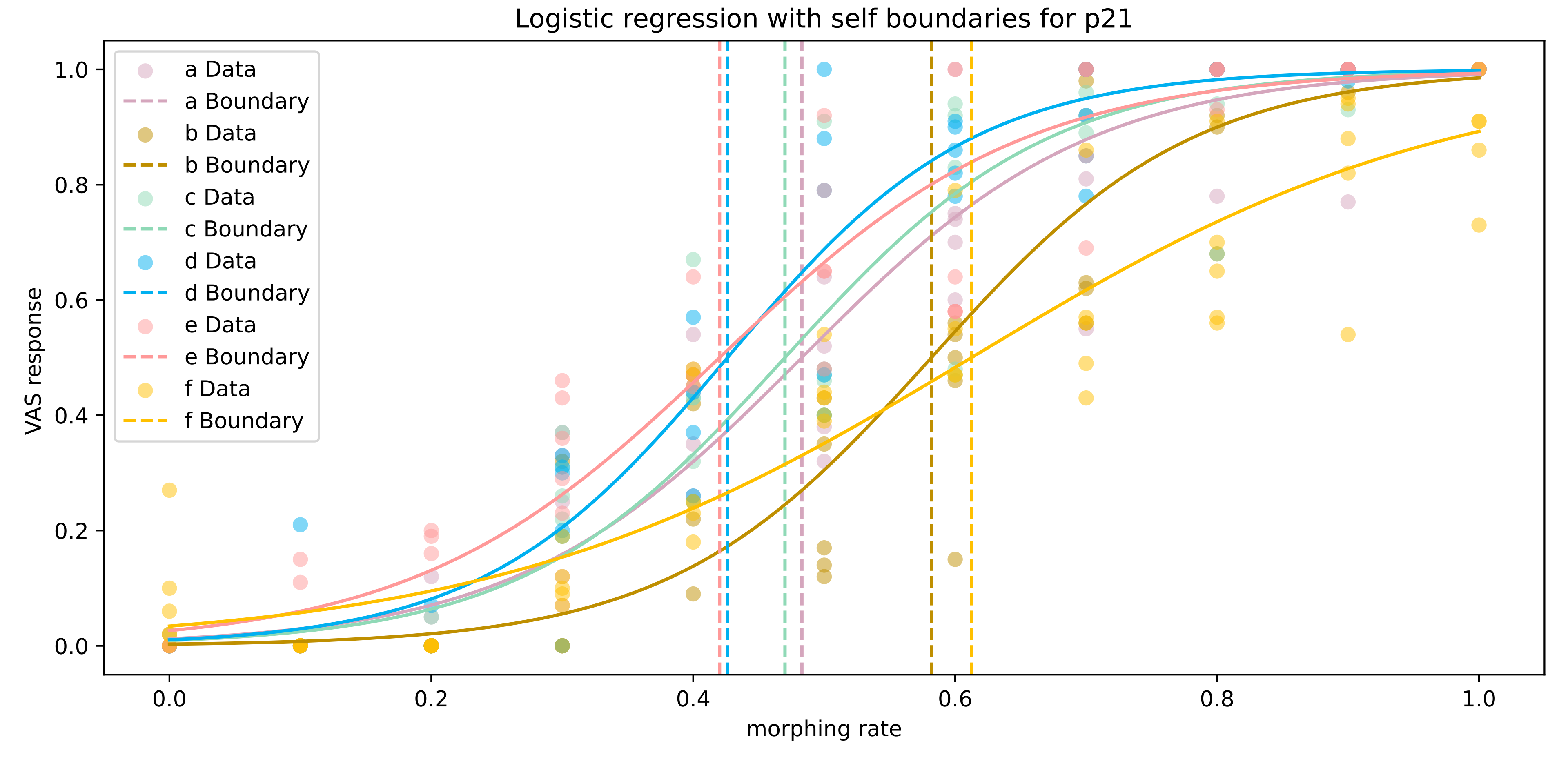

Logistic regression with self boundaries for p21
a Data
a Boundary
b Data
b Boundary
c Data
c Boundary
d Data
d Boundary
e Data
e Boundary
f Data
f Boundary
VAS response
morphing rate
0.0
0.2
0.4
0.6
0.8
1.0

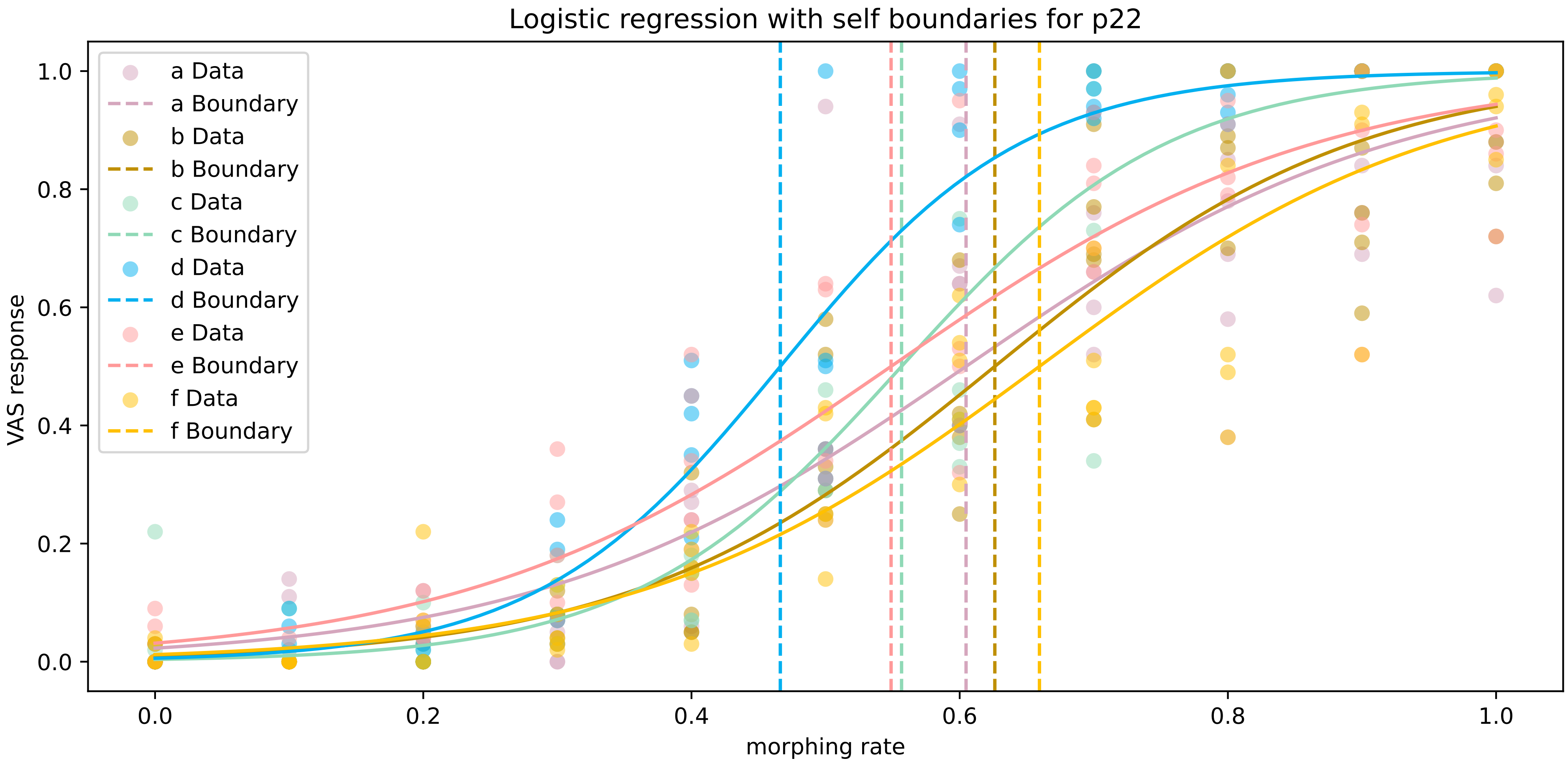

Logistic regression with self boundaries for p22
a Data
a Boundary
b Data
b Boundary
c Data
c Boundary
d Data
d Boundary
e Data
e Boundary
f Data
f Boundary
VAS response
morphing rate
0.0
0.2
0.4
0.6
0.8
1.0

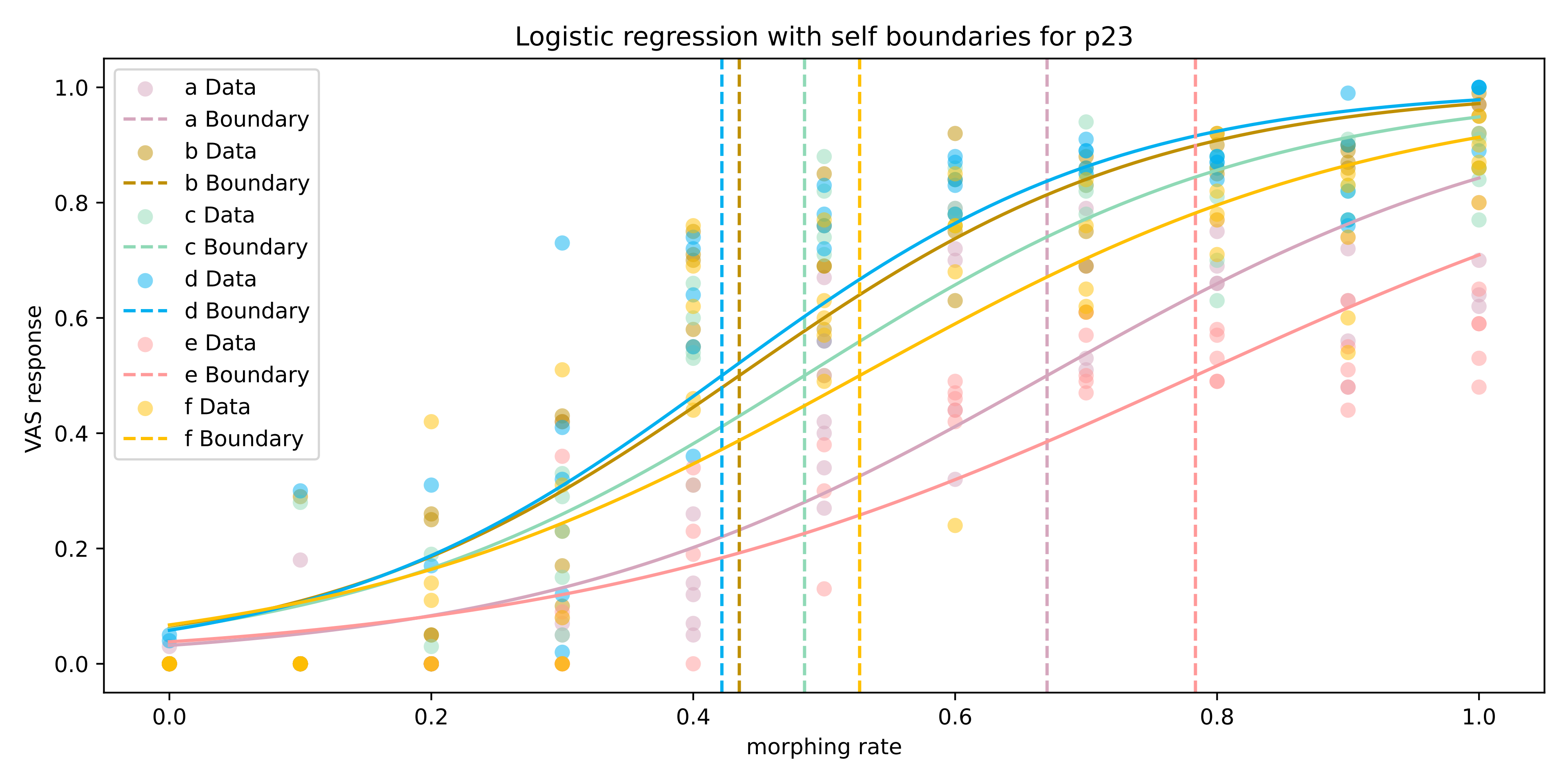

Logistic regression with self boundaries for p23
a Data
a Boundary
b Data
b Boundary
c Data
c Boundary
d Data
d Boundary
e Data
e Boundary
f Data
f Boundary
VAS response
morphing rate
0.0
0.2
0.4
0.6
0.8
1.0

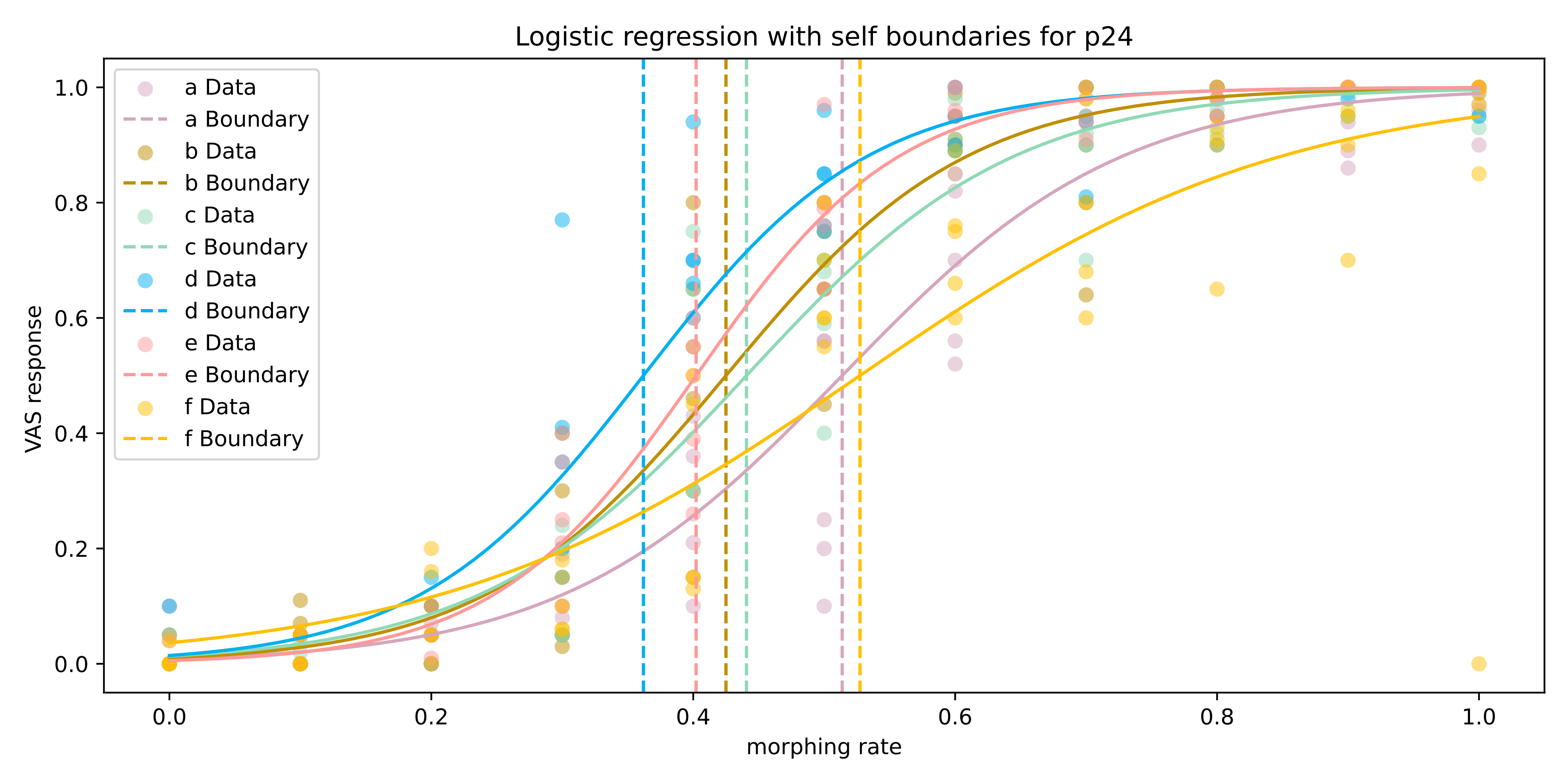

Logistic regression with self boundaries for p24
a Data
a Boundary
b Data
b Boundary
c Data
c Boundary
d Data
d Boundary
e Data
e Boundary
f Data
f Boundary
VAS response
morphing rate
0.0
0.2
0.4
0.6
0.8
1.0

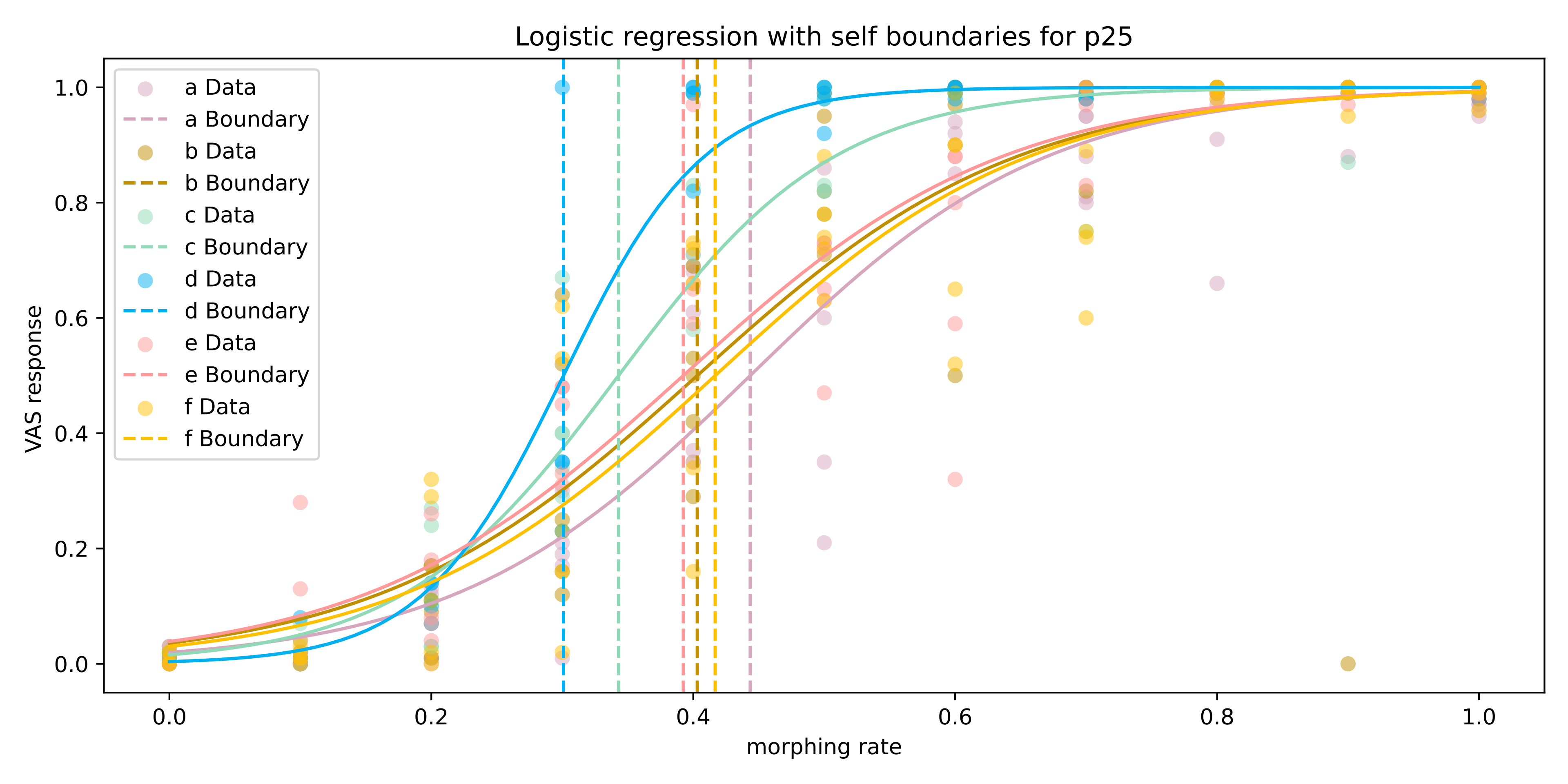

Logistic regression with self boundaries for p25
VAS response
morphing rate
a Data
a Boundary
b Data
b Boundary
c Data
c Boundary
d Data
d Boundary
e Data
e Boundary
f Data
f Boundary
0.0
0.2
0.4
0.6
0.8
1.0

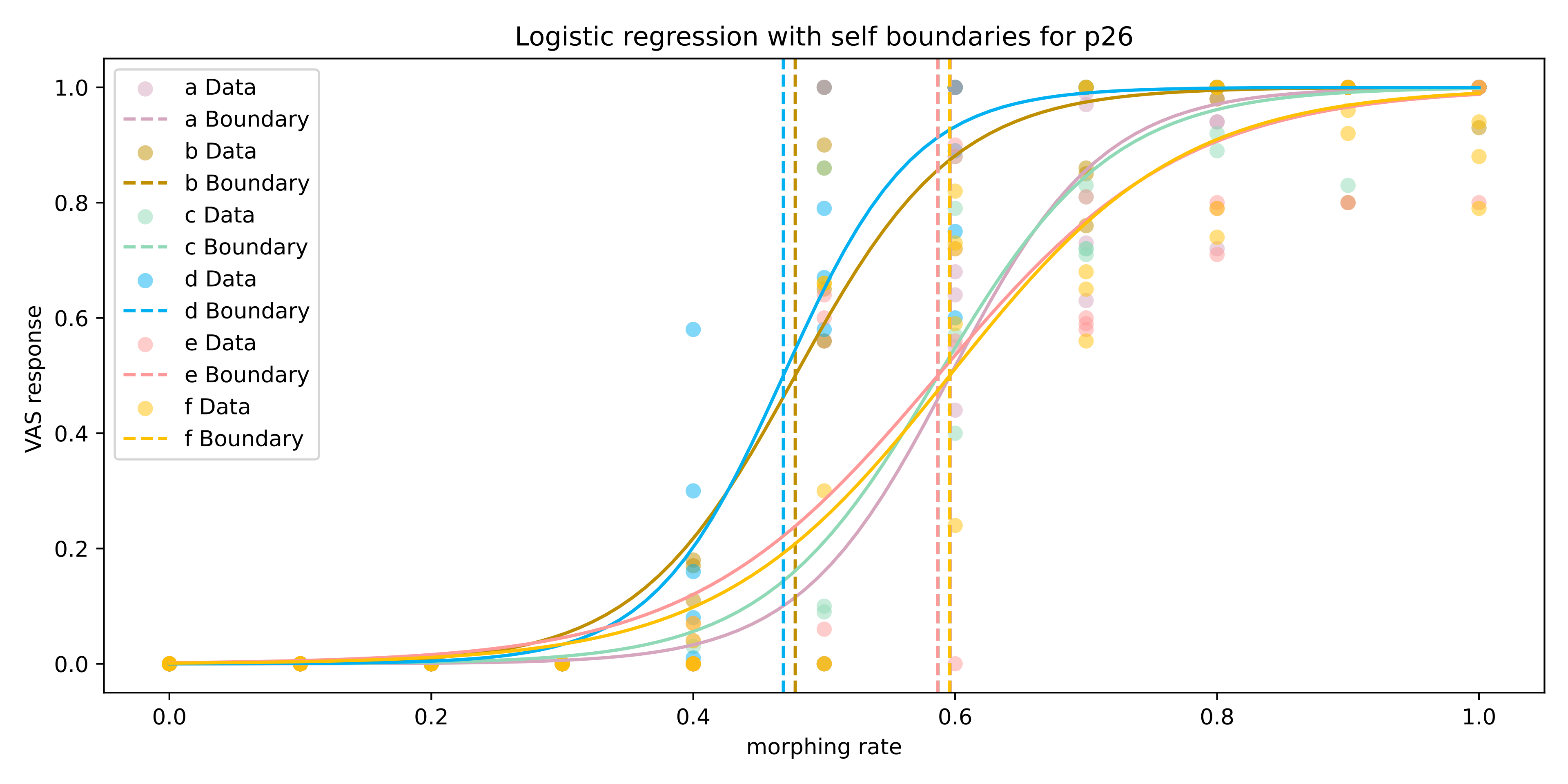
Logistic regression with self boundaries for p26
a Data
a Boundary
b Data
b Boundary
c Data
c Boundary
d Data
d Boundary
e Data
e Boundary
f Data
f Boundary
VAS response
0.0
0.2
0.4
0.6
0.8
1.0
morphing rate
0.0
0.2
0.4
0.6
0.8
1.0

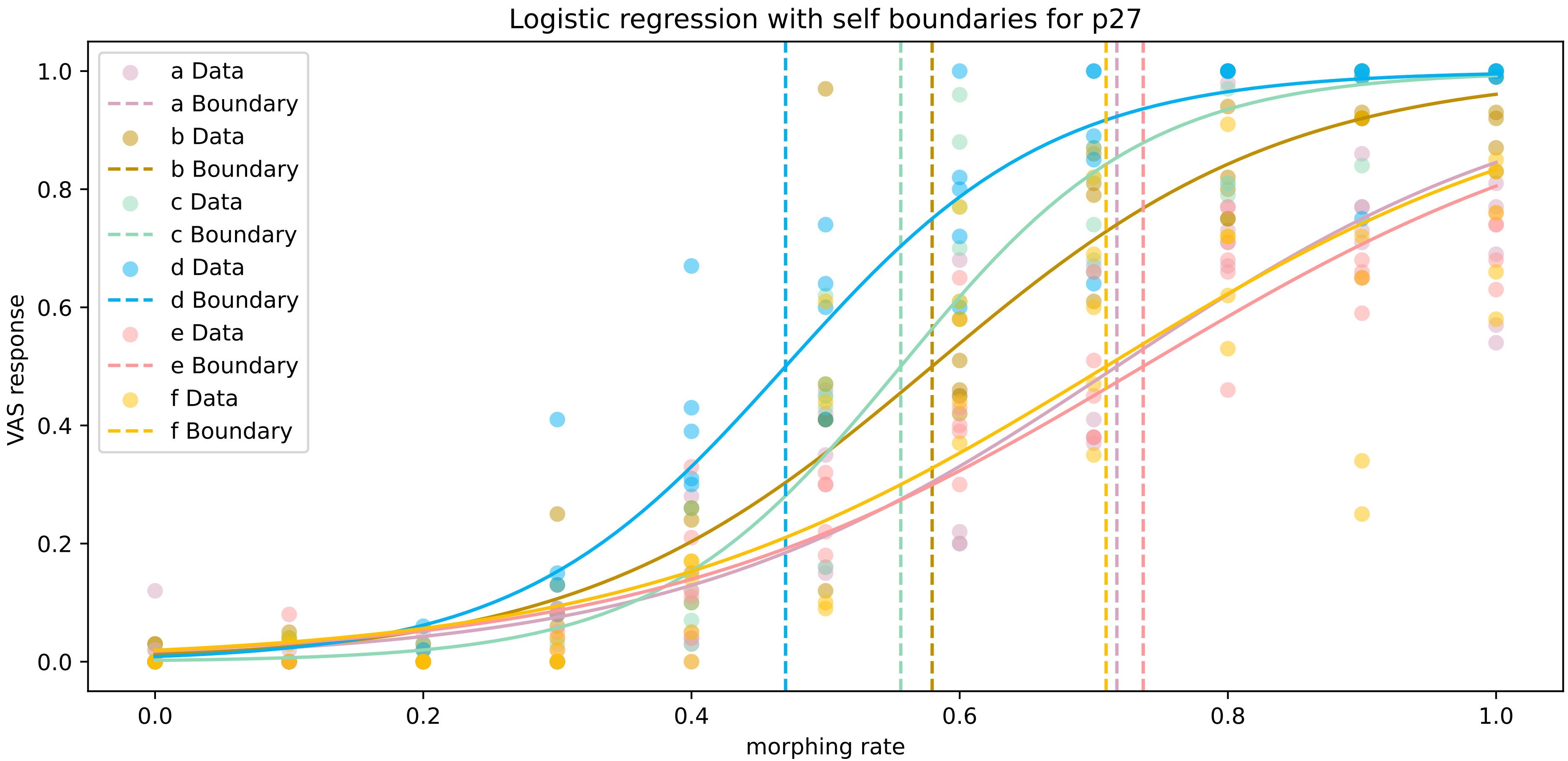

Logistic regression with self boundaries for p27
a Data
a Boundary
b Data
b Boundary
c Data
c Boundary
d Data
d Boundary
e Data
e Boundary
f Data
f Boundary
VAS response
0.0
0.2
0.4
0.6
0.8
1.0
morphing rate
0.0
0.2
0.4
0.6
0.8
1.0

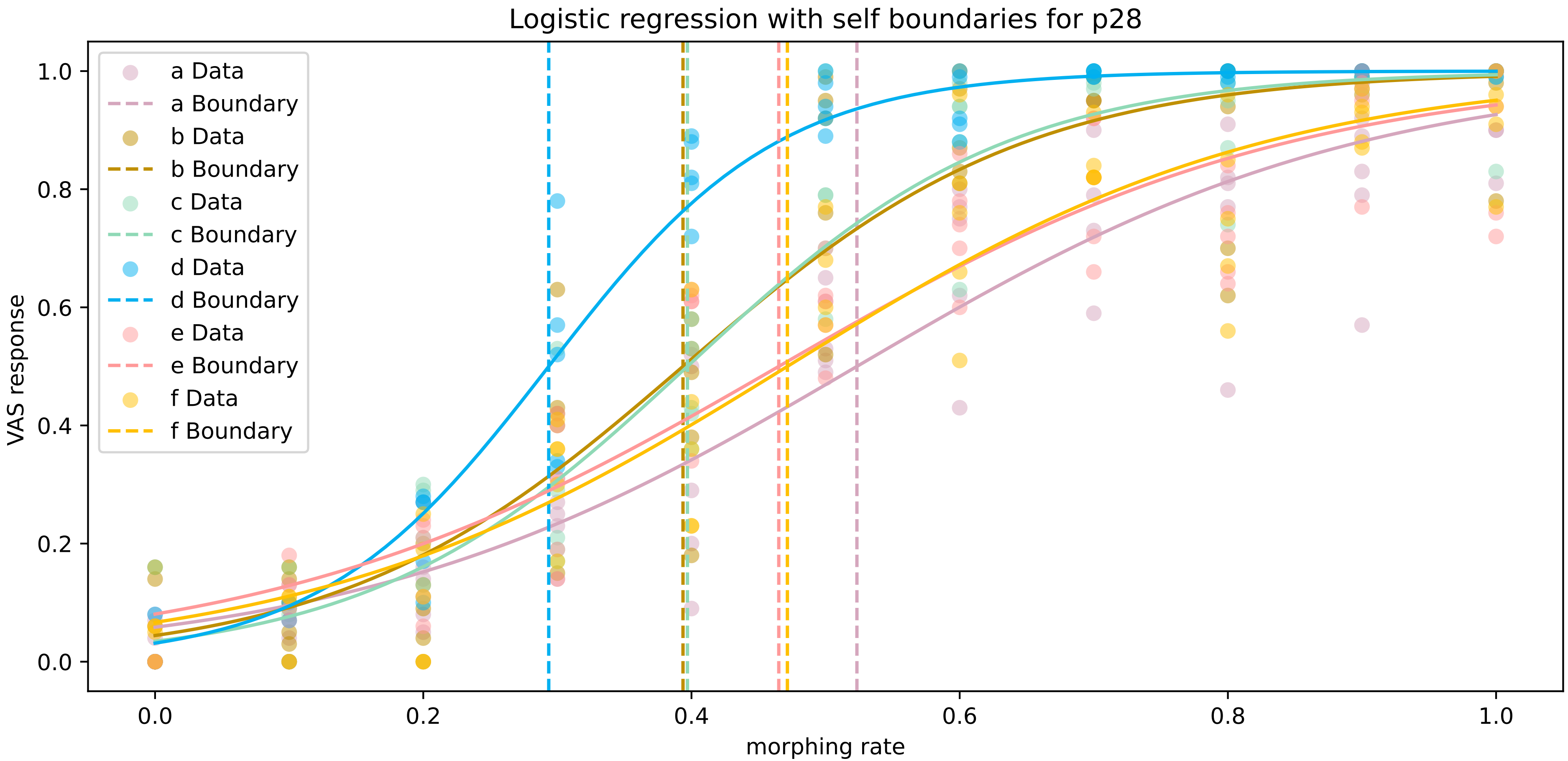

Logistic regression with self boundaries for p28
a Data
a Boundary
b Data
b Boundary
c Data
c Boundary
d Data
d Boundary
e Data
e Boundary
f Data
f Boundary
VAS response
morphing rate
0.0
0.2
0.4
0.6
0.8
1.0

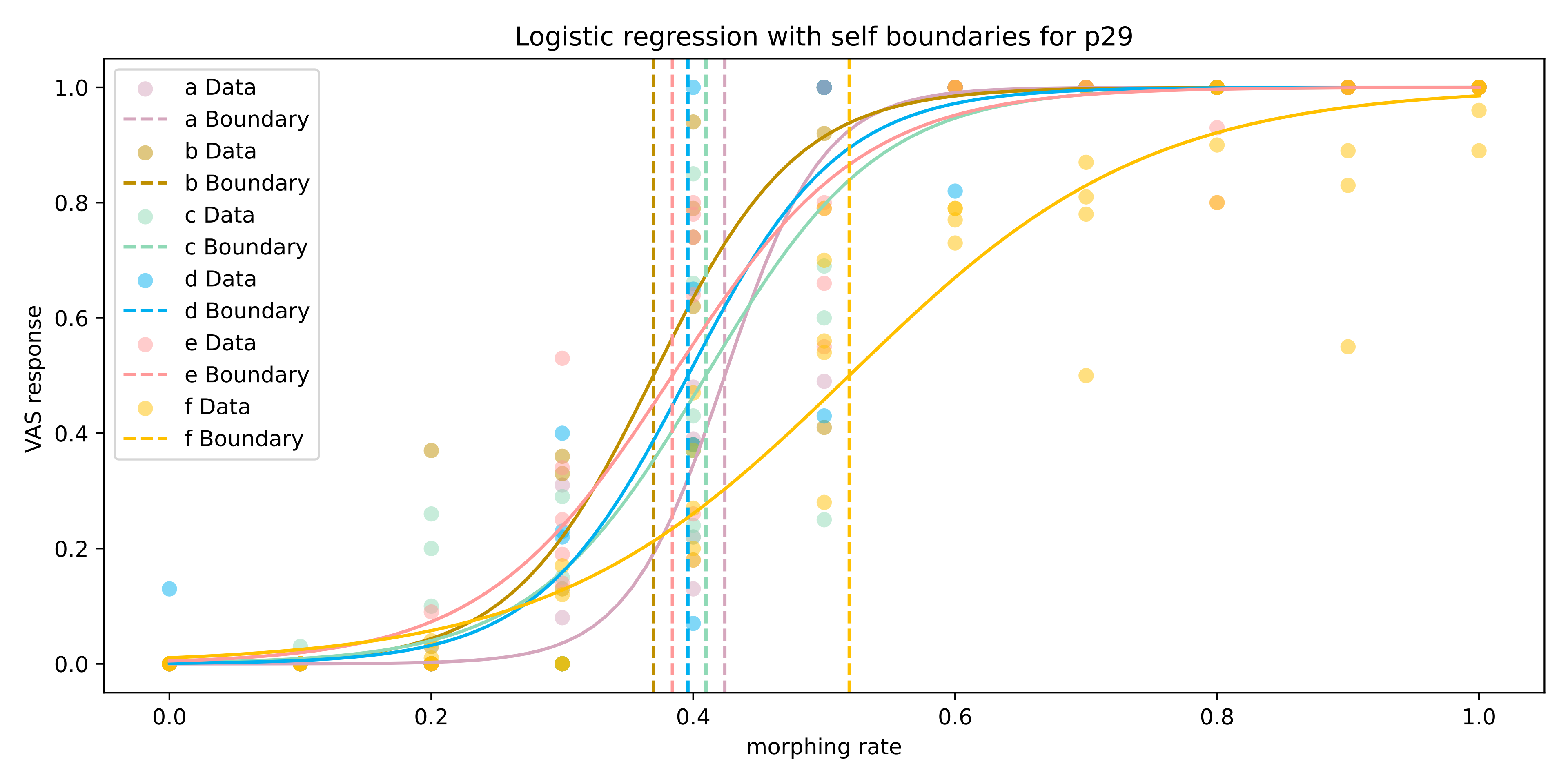

Logistic regression with self boundaries for p29
a Data
a Boundary
b Data
b Boundary
c Data
c Boundary
d Data
d Boundary
e Data
e Boundary
f Data
f Boundary
VAS response
morphing rate
0.0
0.2
0.4
0.6
0.8
1.0

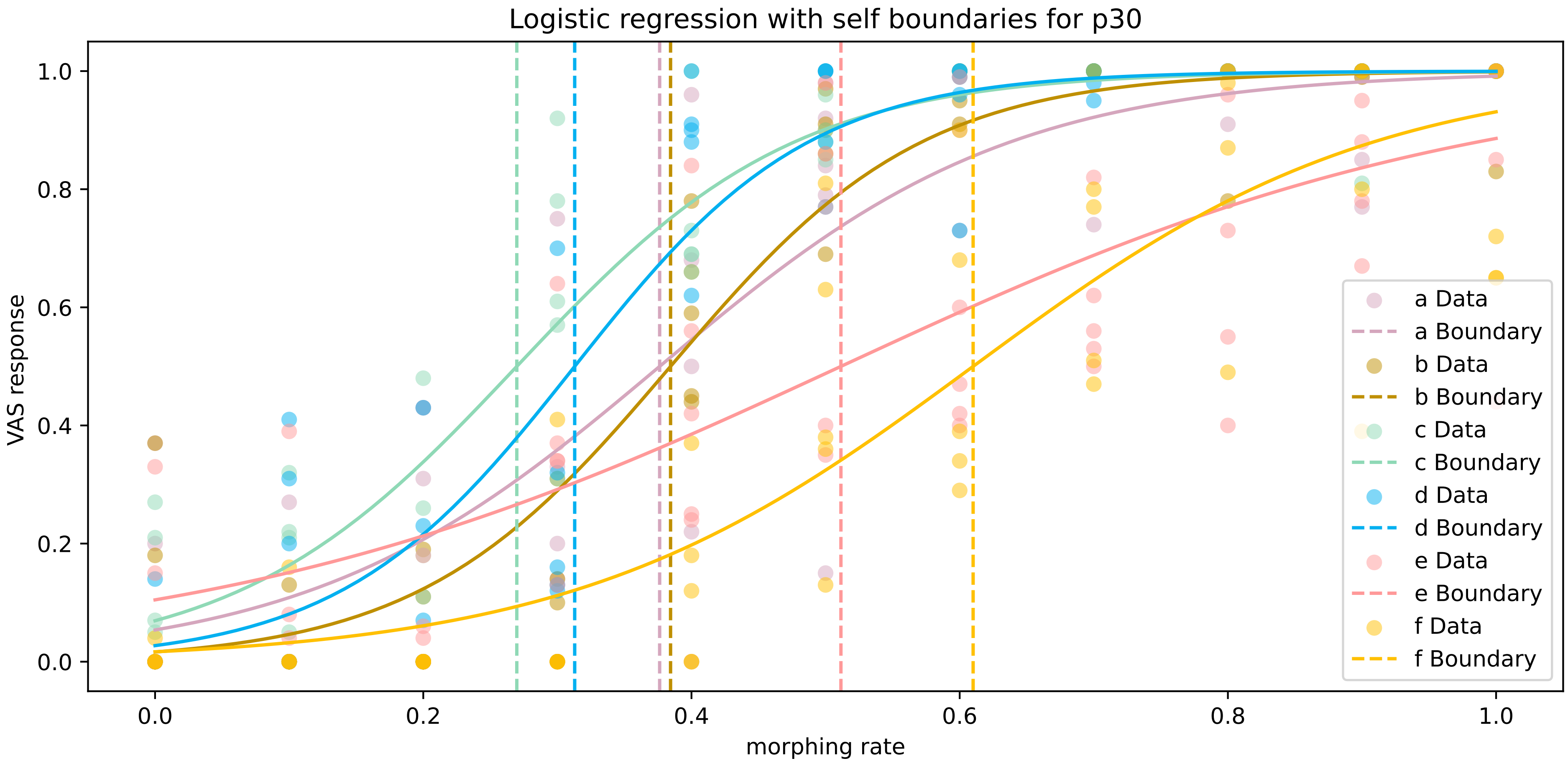
Logistic regression with self boundaries for p30
VAS response
morphing rate
0.0
0.2
0.4
0.6
0.8
1.0
a Data
a Boundary
b Data
b Boundary
c Data
c Boundary
d Data
d Boundary
e Data
e Boundary
f Data
f Boundary
